# Seasonal and daily shifts in behavior and resource selection: how a carnivore navigates costly landscapes

**DOI:** 10.1007/s00442-020-04754-1

**Published:** 2020-09-16

**Authors:** E. Hance Ellington, Erich M. Muntz, Stanley D. Gehrt

**Affiliations:** 1 School of Environment and Natural Resources, Ohio State University, 210 Kottman Hall, 2021 Coffey Road, Columbus, OH 43210 USA; 2 Present Address: Range Cattle Research and Education Center, Wildlife Ecology and Conservation, University of Florida, 3401 Experiment Station Road, Ona, FL 33865 USA; 3 Cape Breton Highlands National Park, PO Box 158, Chéticamp, NS B0E1H0 Canada

**Keywords:** Cape Breton Highlands National Park, Coyote (*Canis latrans*), Home range, Movement, Season, Snow

## Abstract

The dynamic environmental conditions in highly seasonal systems likely have a strong influence on how species use the landscape. Animals must balance seasonal and daily changes to landscape risk with the underlying resources provided by that landscape. One way to balance the seasonal and daily changes in the costs and benefits of a landscape is through behaviorally-explicit resource selection and temporal partitioning. Here, we test whether resource selection of coyotes (*Canis latrans*) in Cape Breton Highlands National Park, Nova Scotia, Canada is behaviorally-explicit and responsive to the daily and seasonal variation to presumed costs and benefits of moving on the landscape. We used GPS data and local convex hulls to estimate space use and Hidden Markov Models to estimate three types of movement behavior: encamped, foraging, and traveling. We then used integrated step-selection analysis to investigate behaviorally explicit resource selection across times of day (diurnal, crepuscular, and nocturnal) and season (snow-free and snow). We found that throughout the day and seasonally coyotes shifted foraging behavior and altered behavior and resource choices to avoid moving across what we could be a challenging landscape. These changes in behavior suggest that coyotes have a complex response to land cover, terrain, and linear corridors that are not only scale dependent but also vary by behavior, diel period, and season. By examining the resource selection across three axes (behavior, time of day, and season), we have a more nuanced understanding of how a predator balances the cost and benefits of a stochastic environment.

**Electronic supplementary material:**

The online version of this article (10.1007/s00442-020-04754-1) contains supplementary material, which is available to authorized users.

## Introduction

Animals use space such that they acquire sufficient resources to meet energetic demands, successfully reproduce, shelter from predation, or harsh environmental conditions, and respond to inter- and intra-specific competition. As such, the structure and composition of the landscape can have a significant influence on the movement and behavior of terrestrial animals, as some might preferentially select resource-rich areas and avoid risky landscapes. The perceived costs and benefits of particular environments can vary both spatially and temporally, especially in highly seasonal systems. For example, light conditions can alter predation risk (Palmer et al. 2017) and energetic costs of movement can increase with seasonal snow cover (Crête and Larivière 2003). Carnivores, particularly apex predators, have a fundamentally different relationship with their environment than other species because they are not directly reliant on vegetation to meet their energetic demands and, excluding human exploitation, predation risk to large carnivores is often not a strong limiting factor.

In temperate climates, the season when the landscape is snow-covered offers challenges for many species and rewards for a few. For example, snow increases the energetic cost of movement (Crête and Larivière 2003) and for many species, subsequently increases predation risk by decreasing their ability to escape. For primary consumers, forage can become less accessible when it is buried under snow. Apex predators, however, can experience increased hunting success if snow makes prey more accessible, for example deep snow might increase the vulnerability of deer to coyote predation (Patterson and Messier 2000). Further, denning, or burrowing species can use snow to create shelter in landscape features that might have been less hospitable during the non-snowy season. The costs and benefits of snow can be modified by the underlying landscape. For example, snow tends to be deeper at higher elevations and on open landscapes, which might increase the energetic cost of movement for many species, whereas snow can be more packed on roads and trails, decreasing the relative cost of movement (Crête and Larivière 2003). Indeed, Thibault and Ouellet (2005) found that coyotes selected open landscapes when snow conditions were favorable but selected forested landscapes when snow conditions were unfavorable.

Dynamic environmental conditions also occur due to the daily cycle of light (dark, twilight, and light). Decreasing light can limit an individual’s ability to perceive the environment, and predators may exploit this limitation during twilight to improve hunting success (Broekhuis et al. 2014). Yet as darkness encroaches, an individual’s ability to effectively move across the landscape can become limited. For example, moving on steep slopes can be risky because of potential injury due to missteps or falling, and dense vegetation might be more difficult to navigate, which can increase the energetic movement cost relative to open landscapes. Predators must navigate the cost:benefit ratio of light (potential injury and increased energetic demand with increased hunting success) as it varies on the landscape. For predators, we might expect that the cost:benefit ratio of snow and the cost:benefit ratio of light vary depending on the underlying behavior—when traveling is the main goal, the predator should seek to minimize the costs of moving on challenging terrain but when hunting is the main goal, the predator should seek to maximize hunting success irrespective of underlying terrain.

The coyote (*Canis latrans*) is a behaviorally plastic carnivore that has successfully colonized most of North America over the last 125 years (Hody and Kays 2018). Where wolves (*Canis lupus*) and large felids (*Panthera onca* and *Puma concolor*) are not present, the coyote is considered an apex predator because they occupy the highest trophic position and have no natural predators. Coyotes are widespread in eastern Canada; however, genetic bottlenecks likely occurred as coyotes expanded eastward, particularly as they colonized islands (Cape Breton, Prince Edward Island, and Newfoundland; Power et al. 2015). Cape Breton Island is characterized by sharp changes in the elevation between deciduous lowlands and a high elevation central plateau of boreal forest and taiga vegetation. Here we used coyotes on Cape Breton Island to examine how animals dynamically modify space use and behavior to account for seasonal or daily changes in risky or costly landscapes. We tested the hypothesis that predator response to the cost:benefit ratio of snow and of light varies depending on the underlying behavior. Specifically, we predicted that when traveling, coyotes will: (1) limit use of areas with high elevation and open landscapes during the snowy season; and (2) limit use of areas with high slope or dense forest in darkness. Conversely, we predicted that the landscape selection patterns of foraging coyotes will vary seasonally, possibly following patterns of forage availability but will not be responsive to seasonal and daily shifts in landscape risk.

## Methods

### Study area

**Fig. 1:**
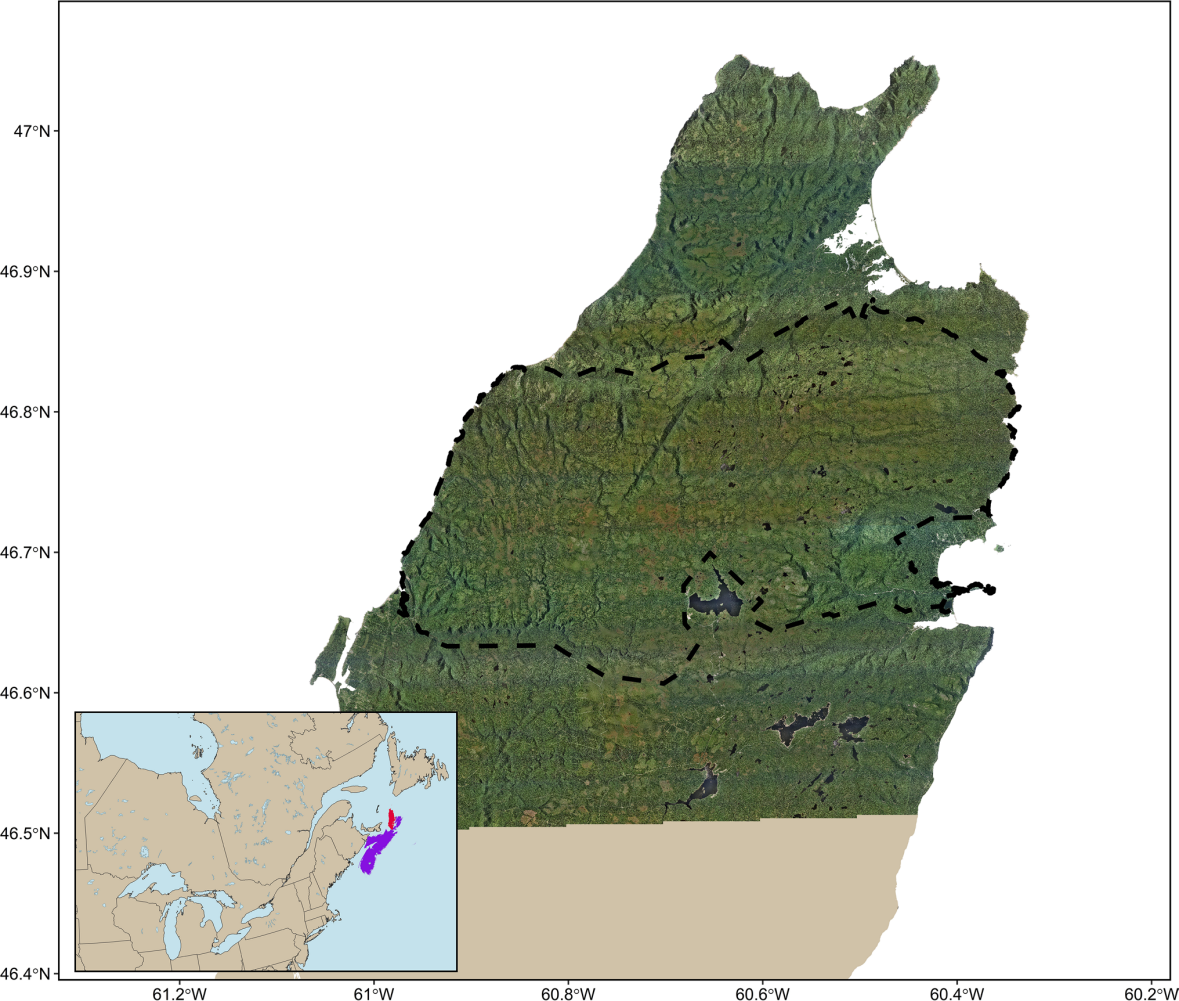
Cape Breton Island, Nova Scotia, Canada where our study was conducted. Most coyotes (*Canis latrans*) were captured within Cape Breton Highlands National Park (black dashed line)

Cape Breton Highlands National Park (CBHNP) is a 948 km^2^ park situated at the north end of Cape Breton Island, Nova Scotia, Canada (Fig. 1). The park is characterized by sharp increases in topography from the park boundaries and canyons to a high central plateau. The central plateau is primarily boreal forest and associated vegetation types, while the lowlands are primarily deciduous Acadian forest. Human disturbance is relatively low in and around the park: a few small towns (< 5000 population) are present near park boundaries, and one major road (three lane highway) runs along three edges of the park. Otherwise, human disturbance is limited to trails and minor roads with low traffic volume that see the majority of use during the snow-free months (June to October). Winters are long in CBHNP and snow cover is typical from December 10 to April 15 at low elevations (< 300 m) and from November 20 to May 1 at high elevations (> 300 m).

Coyotes became established on Cape Breton Island, Nova Scotia, Canada, in the 1980s (Parker 1995). Previous research found the most dominant food items for coyotes in Cape Breton were white-tailed deer (*Odocoileus virginianus*) and snowshoe hare (*Lepus americanus*), while small mammals such as red-backed vole (*Myodes gapperi)*, masked shrew (*Sorex cinereus*), and deer mouse (*Peromyscus maniculatus*) and fruit were also common (Patterson et al. 1998). Coyotes have been documented to prey on moose (Benson and Patterson 2013), but it is unclear to what extent this occurs in CBHNP. Power et al. (2020) found that coyotes in Cape Breton Highlands National Park consumed moose (likely scavenged) year-round, but primarily consumed white-tailed deer during the winter and spring. Potential competitors for coyotes in CBHNP include black bear (*Ursus americanus*), bobcat (*Lynx rufus*), and Canada lynx (*Lynx canadensis*).

### Animal capture

We trapped coyotes in CBHNP from October 2011 to September 2015 using foothold traps or cable restraints. Once captured, we immobilized animals with Telazol, collected physical metrics, and outfitted them with GPS collars (Lotek 7000; Lotek Wireless Inc., Canada or GSC Pinnacle LITE Iridium; Sirtrack, New Zealand). We released animals at capture sites after they recovered from the immobilization. The fix rate schedule of the GPS collars varied between every 2, 6, or 7 h, and occasionally CBHNP personnel increased the fix rate schedule (< 1 h) for various management needs. We removed erroneous fix locations by investigating all locations that resulted in movement speeds greater than 10 km/h. If the locations led to impossible movement speeds (> 50 km/h) or improbable movements based on an individual’s home range and movement patterns, we removed the problematic location. All procedures were conducted in accordance with Parks Canada Research Permit 12020, which was approved by Parks Canada’s Animal Care Committee and followed the guidelines of the American Society of Mammalogists (Sikes and the Animal Care and Use Committee of the American Society of Mammalogists 2016).

### Space use and movement behavior

Using a combination of field observations (including snow tracking), plotting net-squared displacement over time, and manual investigation of space use patterns from the GPS location data, we assigned each individual to one or more space use strategies (see Online Appendix 1 for more details). While historically coyote space use strategy has been based on a binary system (resident and transient; Andelt 1985; Gese et al. 1988; Kamler and Gipson 2000), researchers have recently recognized three distinct space use strategies, although often with different descriptive terms (Morin and Kelly 2017; Sasmal et al. 2019). Here, we use a trinary classification scheme that is most similar to Morin and Kelly (2017). We defined residents as animals that maintained or lived within a territory. Residents could have been either active breeders or members of a group of inter-related individuals which included a breeding pair. We defined local transients (also described as residents displaying biding behavior by Morin and Kelly 2017) as animals that used an area that overlapped one or more resident coyote territories. We defined long-distance transients (also described as transients by Morin and Kelly 2017) as animals that rarely returned to previously used sites during the monitoring period. To accurately assign an individual to a space use strategy we required consecutive locations for a temporal period > 60 days. Over the monitoring period, some individuals switched space use strategies from local transiency to residency (*n* = 2), from residency to local transiency (*n* = 1), and some individuals shifted where their home range occurred on the landscape, but remained residents (*n* = 2).

We estimated home range size of resident and local transient coyotes using the 95% isopleth of the adaptive local convex hull method (LoCoH; Getz et al. 2007). To allow comparisons to other coyote space use studies we also calculated the 95% minimum convex polygon (MCP; Mohr 1947). To reduce the bias on home range estimation from the variable fix rates, we rarified all location data to the longest fix rate present in the data (7 h) using the adehabitatLT package (Calenge 2006) in program R (Core Team R 2019). We generated LoCoH isopleths using the adaptive method and estimated the adaptive sphere of influence (“a”) as the maximum distance between any two locations in the data set (Getz et al. 2007). We generated all home range isopleths using the adehabitatHR package (Calenge 2006) in program R. Coyote territories tend to remain spatially static across seasons (Grinder and Krausman 2001; Gehrt et al. 2009). However, given the highly seasonal landscape of CBHNP, we estimated coyote home ranges both seasonally (snow and snow free) and using the full monitoring period.

To estimate coyote movement behavior (encamped, foraging, and traveling), we rarified all location data with a fix rate of 2 h or less to an approximate 2-h fix rate (rounding to the nearest 15-min interval). We then subset the location data such that we retained at least 8 days of 2-h fixes with no more than 4 consecutive missed fixes at any point in each burst. Using these criteria, our sample included 42 bursts of GPS location data from 15 individuals, ranging from 9 to 76 days in length (Online Appendix 2). We used the adehabitatLT package (Calenge 2006) in R for movement burst assessment and manipulation. Each burst represents a movement path with one step every 2 h in the movement path. Because we had some missing fixes (avg = 8.5%, range 1.3–20.5%), we used a continuous-time correlated random walk model to predict missing locations equivalent to single imputation; (Johnson et al. 2008). We then used the bivariate time series of step lengths and turning angles in this data set to generate movement models based on 2, 3, and 4 movement behavior states with Hidden Markov models (HMM; Michelot et al. 2016) using the R package momentuHMM (McClintock and Michelot 2018). We modeled step lengths using a gamma distribution and modeled turning angles using a wrapped Cauchy distribution. Following Ellington and Gehrt (2019), we assumed the 2-state movement model would delineate two movement behaviors: encamped and moving. Encamped behavior was characterized by short step lengths and high turning angles, and the moving behavior was characterized by long step lengths and low turning angles. We assumed that the 3-state movement model would delineate encamped behavior and further classify moving behavior into foraging, characterized by intermediate step lengths and high turning angles, and traveling, characterized by long step lengths and low turning angles. Finally, we assumed that the 4-state movement model would identify the encamped and traveling movement behaviors, and further distinguish searching behavior, characterized by intermediate step lengths and low turning angles, from foraging behavior, characterized by intermediate step lengths and high turning angles.

Based on the plausible biological interpretation of 2, 3, and 4 movement behavior states, we generated a series of potential step lengths and turning angles to use as starting values for the HMM analysis and ran every combination of these values to ensure that we found the global maximum of the likelihood function (Michelot et al. 2016). We generated predictive movement behavior states for each step (location) using the best models for each number of movement behavior states using the Viterbi algorithm to decode the underlying unobserved Markov chain (Michelot et al. 2016). Finally, for each step (location), we generated the probability of the predicted movement behavior state given the movement model. We then removed any imputed steps (from the continuous‐time correlated random walk model) from further analysis. We assessed model fit and compared the 2-, 3-, and 4-state movement models using three sequential criteria (see Online Appendix 3):

1. Is the model biologically plausible? Here, we examined the movement parameters of each movement state to determine whether the differences in movement behaviors were biologically realistic;
2. Does the model strongly predict individual movement states for each step? Here, we used the average likelihood of the most likely movement state at each movement step as an index of predictive power of the movement model and considered a model strongly predictive if this value was > 0.85.
3. How do biologically plausible and strongly predictive models compare with each other? Here, we used Akaike information criterion (AIC) to compare models.

### Temporal periods and spatial covariates

We assigned each coyote location to a climatic season (snow season [December 10–April 15 at low elevations (< 300 m) and from November 20 to May 1 at high elevations (> 300 m)] or snow-free season) and a light condition (day, crepuscular, night). We estimated light condition by using the position of the sun given the spatial and temporal location using the R package maptools (Bivand and Lewin-Koh 2019); we defined day as 2 h after sunrise to 2 h prior to sunset, night as 2 h after sunset to 2 h prior to sunrise, and the crepuscular period as the two time periods within 2 h of sunrise and sunset.

We estimated vegetation cover using Nova Scotia forest inventory GIS (NS Department of Natural Resources 2016) with unpublished corrections for boreal forest classification (M. Lemieux unpublished). This modified land cover had 10 classes which we reclassified into four classes: forested (alder, Acadian, boreal), open (barren, open intolerant, fern, grass), wetland/freshwater, and anthropogenic. Anthropogenic land cover as defined by the Nova Scotia forest inventory GIS (NS Department of Natural Resources 2016) was rare in our study area and primarily composed of the road network. For vegetation cover, we considered two spatial scales: local (the resolution of the land cover data [10 m]) and landscape (the proportion of a given vegetation cover within a 500 m radius of each location). We also estimated vegetation cover within a 100 m and a 250 m radius of each location, but these measurements were highly correlated with either the local (10 m) or landscape (500 m) scale estimates of vegetation cover. We estimated the forest-open edge density within a 50 m radius of each location. We also estimated landscape heterogeneity within a 500 m radius of each location as the Simpson’s diversity index of forest, open, and wetland. We used the landscape metrics package (Hesselbarth et al. 2019) in R to estimate both edge density and landscape heterogeneity. At each location, we calculated two metrics of landscape terrain generated from the DEM raster (20 m resampled to 10 m resolution; Service Nova Scotia and Municipal Relations 2003): elevation (m) and slope (radians), using the package raster (Hijmans 2019) in program R. Finally, we used the Nova Scotia Roads and Trails dataset (Nova Scotia Geomatics Centre 2014) to identify low use intensity roads (local roads), trails, and utility transmission lines as anthropogenically maintained linear corridors. Because of the ecological similarity for coyotes of low use intensity roads, trails, and utility transmission lines, we combined these features into one class (hereafter, low use intensity corridor). We estimated the density of low use intensity corridors for each location at two spatial scales: local (km/100 m) and landscape (km/500 m) using the packages sp (Bivand et al. 2008) and raster (Hijmans 2019) in program R.

### Resource selection

To evaluate resource selection, we used integrated step selection analysis (iSSA; Avgar et al. 2016). Integrated step selection analysis is an extension on step selection analysis (SSA; Thurfjell et al. 2014) because it allows for the inclusion of movement attributes (step length and turning angles) into the resource selection analysis, thus relaxing the assumption that movement attributes are independent of resource selection. For each observed (used) step, we estimated availability by generating ten random steps from step-length and turn-angle distributions of all individuals using the R package hab (Basille 2015). We characterized each observed and random step with the natural logarithm of step length, the cosine of turning angle, and spatial covariates. We then ran a conditional logistic regression across the different temporal periods (season and time of day) using the R package survival (Therneau 2015). We fit one global iSSA model that contained the spatial covariates forested and open landcover at the local (10 m; binary) and landscape scale (500 m; proportion), forest-open density (50 m; continuous), landscape heterogeneity (500 m; proportion), terrain features at the local scale (elevation [m; 10 m resolution], slope [radians, 10 m resolution], and low use corridor density at local and landscape scales (km/100 m and km/500 m). We considered the land cover type wetland and fresh water as our outgroup (anthropogenic land cover was too rare to adequately function as an outgroup). We estimated the variance inflation factors for the global model for each temporal-behavior dataset using the rms package (Harrell 2020) in R. Across all models, individual variance inflation factors were always < 4.00, indicating that collinearity was unlikely to have a strong influence on our models.

## Results

**Fig. 2:**
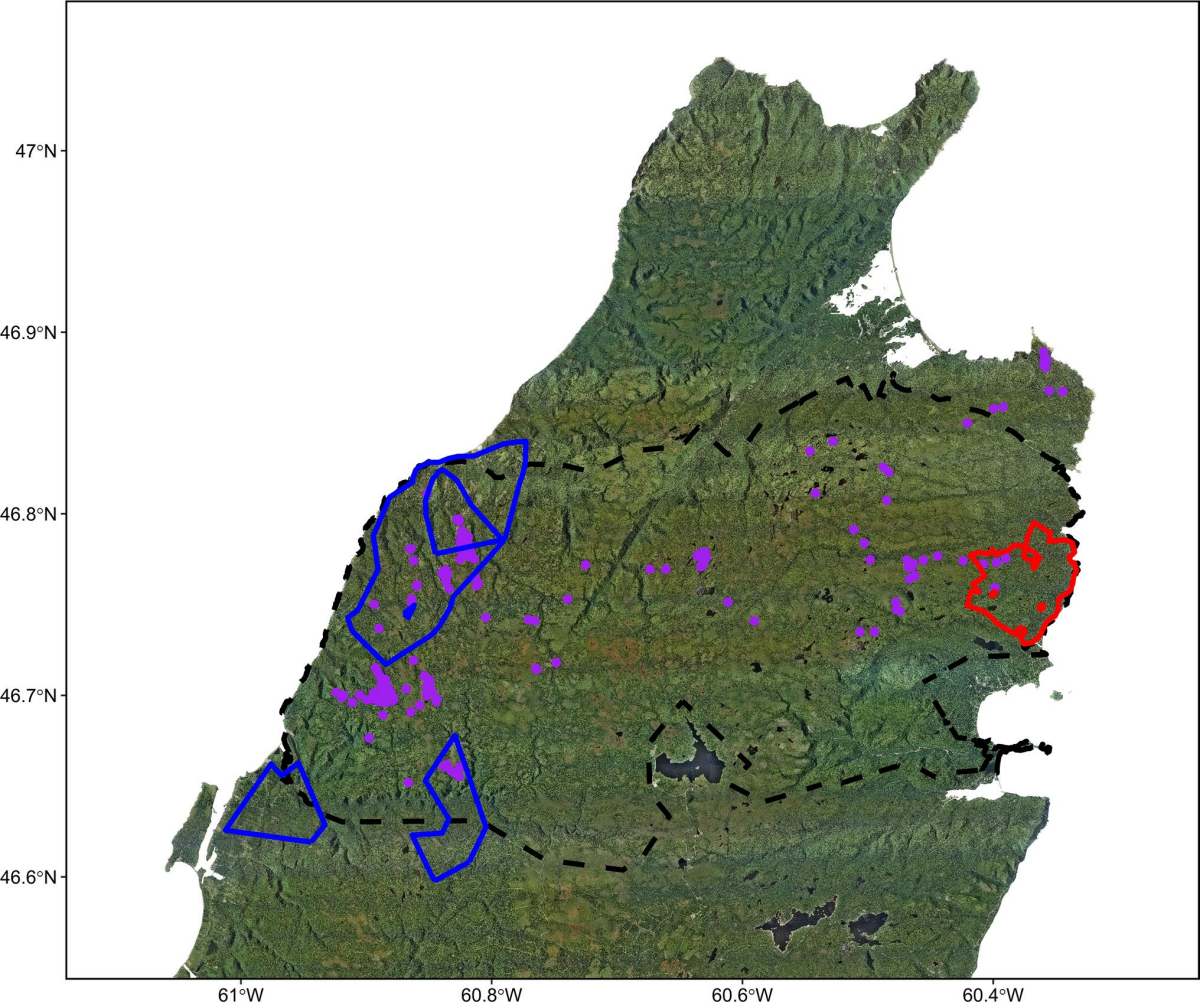
Representative depictions of coyote (*Canis latrans*) space use in and around Cape Breton Highlands National Park, Nova Scotia, Canada (black dashed line). A resident coyote home range (red) and a local transient coyote home range (blue) using the 95% isopleth of the adaptive local convex hull method and the locations of a long-distance transient (purple). Additional home ranges (including seasonal representations) are shown in Online Appendix 2

**Fig. 3:**
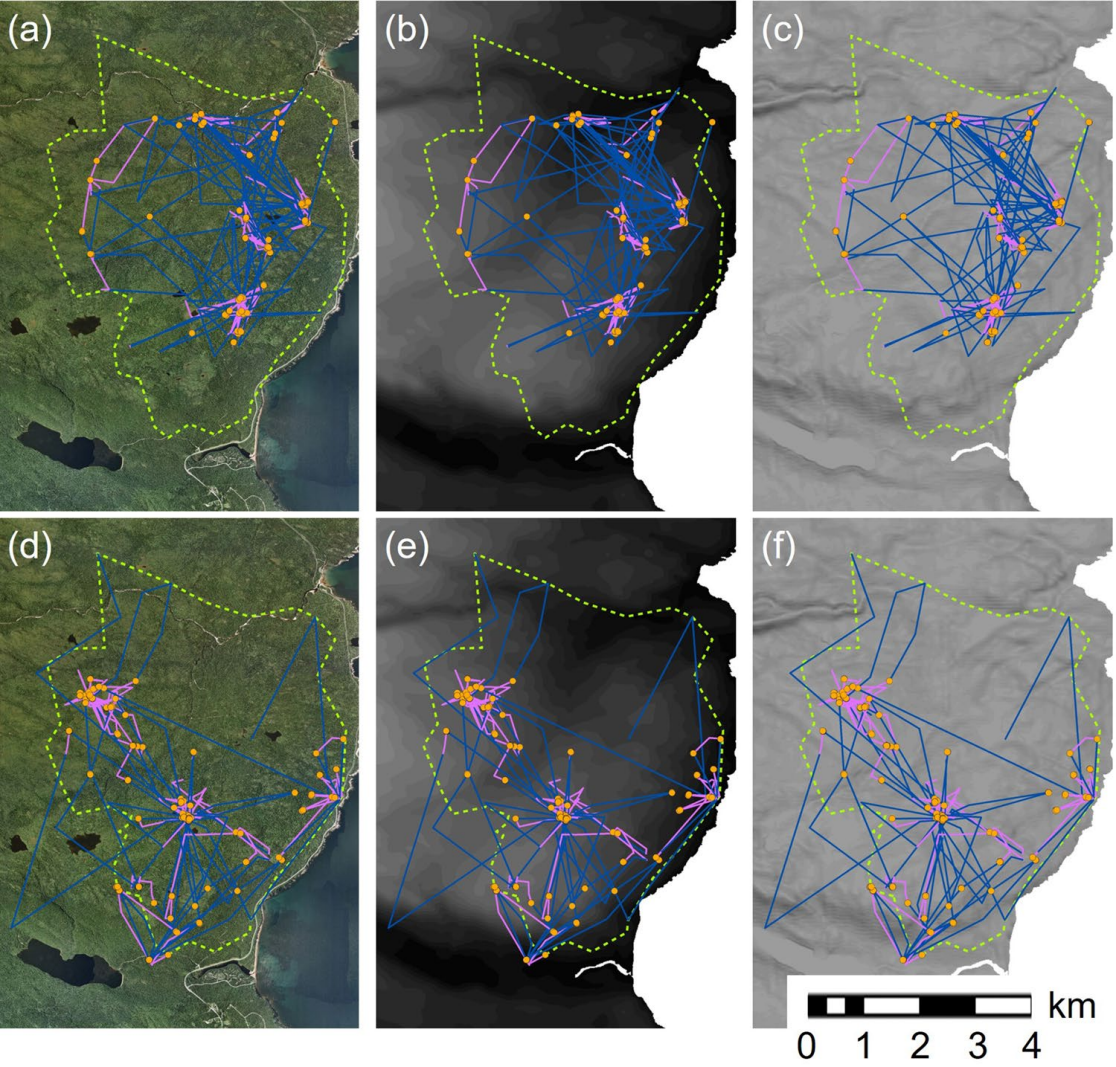
Estimated movement behavior (encamped—orange dots, foraging—pink lines, traveling—blue lines) of a male resident coyote (*Canis latrans*) during the snow-free season (**a**–**c**; 24 May 2014–5 June 2014, 15 June 2014–2 July 2014, and 16 July 2014–2 August 2014) and snow season (**d**–**f**; 17 Nov 2013–7 January 2014) in Cape Breton Highlands National Park, Nova Scotia, Canada. Movement behaviors are mapped onto aerial imagery (**a**, **d**), elevation (low—dark, high—light; **b**, **e**), and slope (shallow—light, steep—dark; **c**, **f**). The 95% isopleth of the adaptive local convex hull polygon is estimated by the dashed green line

**Fig. 4:**
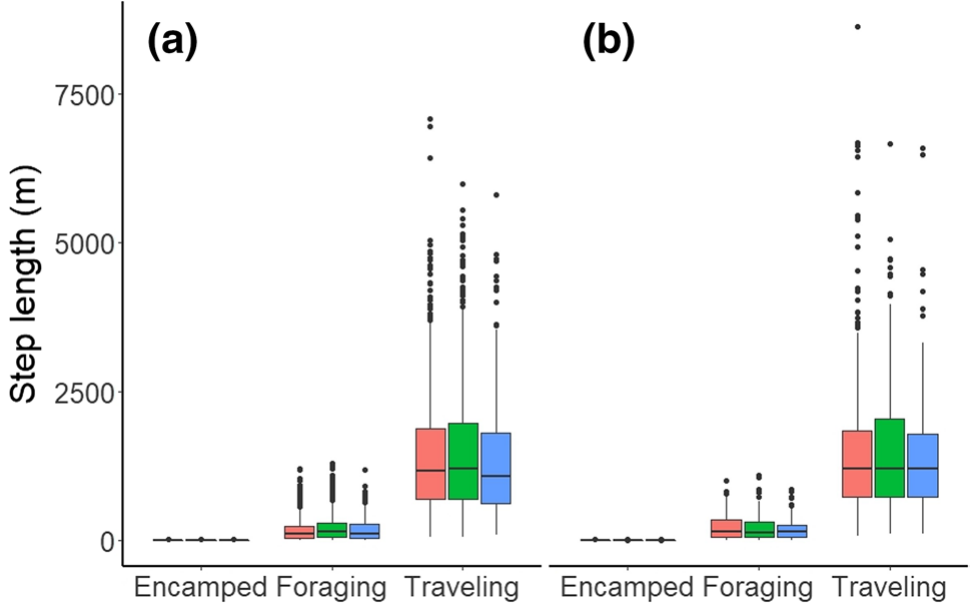
Step lengths of encamped, foraging, and traveling movement behavior of coyotes (*Canis latrans*, *n* = 15) during the diurnal (red), crepuscular (green), and nocturnal (blue) periods during the snow free (**a**) and snow season (**b**) from October 2011 to 2015 in and around Cape Breton Highlands National Park, Nova Scotia, Canada. We estimated movement behavior using hidden Markov models from GPS collars recording locations every 2 h

We captured and collared 17 coyotes (3 females and 14 males). Of these, we used movement paths to classify 14 unique periods of residency, 5 unique periods of local transiency, and 1 period of long-distance transiency. Average home range size of resident coyotes was 26.5 km^2^ (SD = 15.7 km^2^; range 7.5–70.6 km^2^; *n* = 14; Fig. 2, Online Appendix 2). The average home range size of local transient coyotes was 83.9 km^2^ (SD = 25.8 km^2^; range 44.6–113.9 km^2^; *n* = 4; Fig. 2, Online Appendix 2). On average resident coyote home ranges were static seasonally - the average resident home range size during the no snow season was 21.3 km^2^ (SD = 11.5 km^2^; range 5.5–43.5 km^2^, *n* = 10; Online Appendix 2) and during the snow season was 26.2 km^2^ (SD = 19.0 km^2^; range 6.8–60.1 km^2^, *n* = 9; Online Appendix 2). Conversely, the local transient home ranges might not be seasonally static – the average local transient home range size during the no snow season was 52.2 km^2^ (SD = 27.8 km^2^; range 29.5–92.6 km^2^; *n* = 4; Online Appendix 2) and during the snow season was 95.4 km^2^ (range 71.4–119.4 km^2^; *n* = 2; Online Appendix 2). The sole long-distance transient covered a straight-line distance of 48 km and moved at least 273 km over the 66 days it was monitored. We were able to estimate movement behavior from 42 unique bursts of location data from 15 coyotes (e.g., Fig. 3, also see Online Appendix 2). The step lengths of encamped, foraging, and traveling movement behaviors did not differ across seasons or the diel cycle (Fig. 4).

### Encamped

When we classified coyotes as encamped, the average step length was 7 m (range 0–29 m). This result is likely reflective of both short movements during the encamped behavior and small discrepancies in GPS telemetry seen in stationary collars. During the snow-free season coyotes spent 31% of their time encamped (the least frequent behavior; approximately 7.4 h a day), conversely during the snow season coyotes spent 40% of their time encamped (the most frequent behavior; 9.5 h a day) (Table 1). This increase in time spent encamped was entirely driven by an increase in time spent encamped during crepuscular and nocturnal periods (Table 1).

**Table 1:** Proportion of time and estimate hours per day that coyotes (*Canis latrans*; *n* = 15) spent using each movement behavior (encamped, foraging, and traveling) in different light conditions and during different seasons

	Encamped	Foraging	Traveling
Snow-free season			
Diurnal	0.32 (3.18 h, *n* = 1032)	0.36 (3.59 h, *n* = 1165)	0.33 (3.28 h, *n* = 1065)
Crepuscular	0.25 (2.03 h, *n* = 658)	0.32 (2.55 h, *n* = 826)	0.43 (3.39 h, *n* = 1100)
Nocturnal	0.36 (2.15 h, *n* = 698)	0.31 (1.84 h, *n* = 596)	0.33 (1.99 h, *n* = 645)
Snow season			
Diurnal	0.32 (3.18 h, *n* = 274)	0.30 (2.99 h, *n* = 258)	0.38 (3.86 h, *n* = 333)
Crepuscular	0.42 (3.42 h, *n* = 295)	0.27 (2.19 h, *n* = 189)	0.31 (2.48 h, *n* = 214)
Nocturnal	0.50 (2.91 h, *n* = 251)	0.26 (1.51 h, *n* = 130)	0.25 (1.45 h, *n* = 125)

We estimated movement behavior using hidden Markov models from coyotes monitored using GPS collars recording locations every 2 h from October 2011 to October 2015 in Cape Breton Highlands National Park, Nova Scotia, Canada

^a^*n* is the number of locations in this behavior summed over all animals

Land cover did not strongly influence where coyotes were encamped during any time period (Tables 2 and 3).

**Table 2:** Coefficients of resource selection functions for coyote (*Canis latrans*) movement behavior (encamped, foraging, and traveling) during diurnal, crepuscular (crep.), and nocturnal periods in the snow-free season

Variable	Encamped			Foraging			Traveling		
	Diurnal	Crep.	Nocturnal	Diurnal	Crep.	Nocturnal	Diurnal	Crep.	Nocturnal
Forest at 10 m	– 0.12 (0.25)	– 0.44 (0.35)	– 0.08 (0.34)	– 0.03 (0.12)	– 0.14 (0.13)	– 0.28 (0.17)	**– 0.32 (0.13)**	– 0.19 (0.12)	– 0.11 (0.15)
Open at 10 m	– 0.16 (0.29)	– 0.58 (0.40)	– 0.25 (0.37)	**0.41 (0.15)**	0.09 (0.16)	0.13 (0.21)	– 0.13 (0.15)	0.22 (0.15)	**0.53 (0.20)**
Forest at 500 m	0.71 (1.42)	– 0.70 (1.81)	0.75 (2.24)	– 0.21 (0.36)	0.16 (0.40)	– 0.32 (0.49)	**0.92 (0.34)**	0.42 (0.30)	0.44 (0.37)
Open at 500 m	– 0.89 (1.91)	– 0.73 (2.38)	– 0.28 (2.64)	– 0.80 (0.47)	**– 1.50 (0.56)**	**– 1.83 (0.74)**	0.01 (0.39)	– 0.41 (0.39)	**– 1.56 (0.53)**
Forest–open edge density at 50 m	0.20 (0.24)	0.30 (0.34)	0.01 (0.30)	0.05 (0.11)	**0.31 (0.13)**	– 0.10 (0.16)	– 0.10 (0.10)	– 0.06 (0.11)	0.03 (0.15)
Landscape heterogeneity at 500 m^a^	0.39 (0.79)	– 0.33 (1.06)	0.75 (1.28)	0.31 (0.21)	**0.78 (0.26)**	0.17 (0.33)	**0.47 (0.18)**	0.12 (0.18)	**0.73 (0.22)**
Linear corridor density at 100 m	– 0.31 (1.01)	0.60 (1.34)	– 1.00 (1.44)	**– 1.56 (0.43)**	**– 1.42 (0.47)**	**– 1.41 (0.52)**	**– 1.18 (0.43)**	– 0.27 (0.39)	– 0.16 (0.45)
Linear corridor density at 500 m	0.15 (3.84)	4.23 (5.14)	3.22 (5.80)	**3.52 (1.10)**	**2.61 (1.17)**	**4.13 (1.37)**	**3.12 (1.00)**	**2.29 (0.93)**	**2.56 (1.16)**
Elevation (per 100 m)	0.32 (0.32)	0.08 (0.42)	0.19 (0.56)	0.15 (0.08)	0.12 (0.10)	0.07 (0.12)	**0.20 (0.07)**	0.09 (0.06)	**– 0.25 (0.08)**
Slope (radians)	– 0.43 (1.18)	– 0.06 (1.57)	– 0.37 (1.90)	– 0.29 (0.39)	**– 1.05 (0.49)**	**– 2.65 (0.64)**	**– 1.84 (0.42)**	**– 2.98 (0.41)**	**– 2.66 (0.53)**
Step distance (m, natural log)	**– 0.71 (0.03)**	**– 0.77 (0.04)**	**– 0.79 (0.04)**	**0.04 (0.01)**	**0.10 (0.02)**	**0.06 (0.02)**	**0.80 (0.03)**	**0.87 (0.03)**	**0.79 (0.04)**
Turning angle (cosine)	**– 0.40 (0.05)**	**– 0.54 (0.07)**	**– 0.47 (0.06)**	0.03 (0.04)	– 0.06 (0.05)	– 0.05 (0.06)	**0.50 (0.05)**	**0.35 (0.05)**	**0.38 (0.06)**

We estimated movement behavior using hidden Markov models from coyotes monitored using GPS collars recording locations every 2 h from October 2011 to October 2015 in Cape Breton Highlands National Park, Nova Scotia, Canada. Significant coefficients (2 × SE < β) are in bold font

^a^Landscape heterogeneity was measured as the Simpson’s Diversity Index between forested, open, and wetland land cover

**Table 3:** Coefficients of resource selection functions for coyote (*Canis latrans*) movement behavior (encamped, foraging, and traveling) during diurnal, crepuscular (crep.), and nocturnal periods in the snow season

Variable	Encamped			Foraging			Traveling		
	Diurnal	Crep.	Nocturnal	Diurnal	Crep.	Nocturnal	Diurnal	Crep.	Nocturnal
Forest at 10 m	– 0.13 (0.47)	0.52 (0.51)	– 0.45 (0.52)	**– 0.71 (0.22)**	– 0.46 (0.25)	– 0.14 (0.32)	– 0.13 (0.20)	0.21 (0.27)	0.55 (0.33)
Open at 10 m	– 0.21 (0.53)	0.58 (0.55)	– 0.79 (0.56)	**– 0.66 (0.26)**	– 0.27 (0.29)	0.02 (0.35)	– 0.07 (0.23)	– 0.05 (0.31)	0.10 (0.39)
Forest at 500 m	0.08 (3.49)	– 0.59 (3.65)	1.38 (3.66)	**1.96 (0.71)**	**1.80 (0.85)**	1.19 (1.12)	0.75 (0.51)	– 0.23 (0.62)	– 0.53 (0.83)
Open at 500 m	– 0.94 (4.31)	0.00 (4.89)	1.98 (4.32)	– 0.04 (0.81)	– 0.93 (1.03)	– 2.32 (1.27)	– 0.90 (0.62)	– 0.70 (0.77)	0.30 (1.06)
Forest-open edge density at 50 m	0.07 (0.34)	– 0.24 (0.43)	– 0.17 (0.43)	0.05 (0.17)	0.08 (0.19)	– 0.38 (0.25)	– 0.00 (0.15)	0.11 (0.19)	– 0.29 (0.24)
Landscape heterogeneity at 500 m^a^	0.25 (2.89)	– 0.12 (2.97)	0.31 (2.67)	0.74 (0.49)	0.90 (0.60)	**1.60 (0.75)**	0.42 (0.37)	– 0.60 (0.45)	0.26 (0.65)
Linear corridor density at 100 m	1.43 (5.13)	0.41 (3.72)	– 3.12 (3.12)	0.53 (1.39)	0.55 (1.32)	– 7.23 (4.72)	0.98 (0.91)	– 0.56 (1.20)	**3.03 (1.47)**
Linear corridor density at 500 m	– 3.79 (16.09)	– 10.45 (17.10)	– 1.89 (16.06)	– 5.01 (3.62)	– 0.88 (3.73)	2.58 (4.77)	– 3.69 (2.38)	1.78 (2.88)	– 8.66 (4.41)
Elevation (per 100 m)	2.07 (1.91)	0.11 (1.95)	1.38 (1.27)	**1.11 (0.35)**	**0.70 (0.30)**	**1.04 (0.42)**	**0.31 (0.15)**	0.29 (0.16)	– 0.13 (0.23)
Slope (radians)	3.18 (4.40)	– 4.63 (5.29)	– 3.70 (3.85)	0.59 (1.04)	– 1.50 (1.16)	0.18 (1.34)	– 1.41 (0.74)	– 1.37 (0.80)	**– 4.38 (1.43)**
Step distance (m, natural log)	**– 0.87 (0.07)**	**– 0.90 (0.07)**	**– 0.89 (0.07)**	**0.10 (0.03)**	**0.09 (0.03)**	**0.09 (0.04)**	**0.82 (0.06)**	**0.87 (0.08)**	**0.98 (0.11)**
Turning angle (cosine)	**– 0.34 (0.10)**	**– 0.49 (0.10)**	**– 0.42 (0.10)**	0.13 (0.09)	0.02 (0.10)	0.03 (0.12)	**0.55 (0.09)**	**0.25 (0.11)**	**0.46 (0.15)**

We estimated movement behavior using hidden Markov models from coyotes monitored using GPS collars recording locations every 2 h from October 2011 to October 2015 in Cape Breton Highlands National Park, Nova Scotia, Canada. Significant coefficients (2 × SE < β) are in bold font

^a^Landscape heterogeneity was measured as the Simpson’s Diversity Index between forested, open, and wetland land cover

### Foraging

When we classified coyotes as foraging, the average step length was 198 m (range 0–1297 m). During the snow-free season, foraging was the second most frequent behavior (33%; 8.0 h a day), but during the snow season it was the least frequent behavior (28%; 6.7 h a day; Table 1). This decline in time spent foraging was relatively equal across time periods (Table 1).

Where coyotes foraged was associated with land cover both seasonally and across time periods. When foraging in the snow-free season, coyotes preferred open patches during the day but always avoided open landscapes in low light conditions (crepuscular and nocturnal) (Table 2). In addition, during the crepuscular time period, foraging coyotes preferred forest-open edge and a more heterogenous landscape (Table 2). When foraging in the snow-free season, the coyote response to linear corridor density was scale dependent: they avoided high densities of linear corridors at the patch level and showed a preference for high densities of linear corridors at the landscape level regardless of the time of day (Table 2). Foraging coyotes also avoided slope during low light conditions (crepuscular and nocturnal) but not during the day (Table 2)—this corresponded to our prediction. During the snow season foraging coyotes responded to the landscape differently than during the snow-free season. In the snow season foraging coyotes preferred forested landscapes (500 m) during the day and during the crepuscular hours (Table 3). Yet, foraging coyotes also avoided both open and forested patches (10 m) during the day during the snow season (Table 3). In addition, during the snow season, foraging coyotes preferred a heterogenous landscape but only at night (Table 3). When foraging in the snow season, coyotes showed no response to linear corridor density at either the patch or landscape level and the only topographic feature foraging coyotes responded to was elevation—they preferred higher elevations regardless of the time of day (Table 3).

### Traveling

When we classified coyotes as traveling, the average step length was 1426 m (range 59–8619 m). During the snow-free season, traveling was the most frequent behavior (36%; 8.7 h a day) and during the snow season, it was the second most frequent behavior (32%; 7.8 h a day; Table 1). However, this decline in traveling behavior was not uniform across the diel cycle, in fact coyotes spent more time traveling during the diurnal period in the snow season than during the diurnal period in the snow-free season (3.3 h a day vs 3.9 h a day; Table 1). Coyotes spent less time traveling during the crepuscular and nocturnal periods during the snow season than during the snow-free season (3.4 h a day vs 2.5 h day during the crepuscular period and 2.0 h a day vs 1.5 h a day during the nocturnal period; Table 1).

During the snow-free season, traveling coyotes avoided forest patches but preferred forested landscapes during the day. Although at night, traveling coyotes avoided open patches but preferred open landscapes (Table 2). During the day and at night traveling coyotes preferred heterogenous landscapes. Yet during crepuscular hours traveling coyotes did not respond to land cover. The response of traveling coyotes to linear corridor density was also scale dependent—during the day in the snow-free season, coyotes avoided high densities of linear corridors at the patch level but preferred them at the landscape level (Table 2). Interestingly, during the crepuscular and nocturnal periods in the snow-free season, coyotes showed no response to linear corridor density at the patch level, but continued to prefer higher densities of linear corridors at the landscape level (Table 2). Topographically, during the snow-free season, traveling coyotes preferred higher elevations during the day and lower elevations at night but regardless of time of day, coyotes avoided steep slopes (Table 2). This avoidance was stronger; however, during the crepuscular and nocturnal periods than during the day, corresponding to our prediction. Conversely, during the snow season, traveling coyotes showed relatively little response to landscape features. During the day, traveling coyotes preferred higher elevations and at night traveling coyotes preferred higher densities of linear corridors at the patch level and avoided slopes (Table 3).

## Discussion

The risks that an animal faces and the rewards that it obtains from its environment can change seasonally and even daily. Even for a carnivore thought to be a generalist, darkness, and snow can affect how an individual uses space and behaves within its environment. By accounting for coyote behavior, the diel cycle, and the seasonal snow cycle, we draw two main conclusions from patterns of coyote resource selection: (1) coyotes shift foraging behavior across both the diel cycle and season; and (2) coyotes alter behavior and resource choices to minimize movement through what could be challenging terrain, and the relative difficulty of that terrain itself varies daily and seasonally.

Beyond the balancing of cost and benefits of a seasonally stochastic environment and the daily shifts in potential risk, it is also important to place our findings in the broader context of coyote ecology. Home ranges of resident coyotes in CBHNP (*x̄* = 26.5 km^2^ LCH; *x̄* = 44.1 km^2^ MCP) were similar in size to those observed outside of the CBHNP on Cape Breton (*x̄* = 49.3 km^2^ [*n* = 8], adaptive kernel method; Patterson and Messier 2001) and were not considerably larger than the average resident coyote home range across eastern North America (*x̄* = 39.5 km^2^ MCP; Ellington and Murray 2015). We expected that coyote home ranges in CBHNP would be similar to those seen in forested landscapes of southeastern Quebec, Canada (Tremblay et al. 1998; Crête et al. 2001), the Gaspesie Provincial Park in eastern Quebec (Boisjoly et al. 2010), or in Newfoundland, Canada (Ellington 2016), yet in these areas coyote home ranges were considerably larger than in CBHNP. Perhaps these differences are driven by our more nuanced approach to identifying coyote space use strategies—local transient coyotes use larger home ranges and if classified as residents, they would have driven the average home range size higher. Similar to Sasmal et al. (2019), we also saw seasonal differences in local transient coyote home ranges, but not in resident home ranges, further buoying the need for home range analysis approaches that consider a trinary classification of space use strategy.

Coyotes in CBHNP spent approximately 1/3 of their time in each behavior (encamped, foraging, and traveling). Coyotes in urban and suburban Chicago, Illinois, USA, however (where average resident home range size was 4.6 km^2^ LCH), only spent about 1/5 of their time foraging and 1/5 traveling, and instead spent 3/5 of their time encamped (Ellington and Gehrt 2019). These differences in how coyotes partitioned behavior could be driven by: (1) coyotes limiting exposure to humans in urban landscapes by spending less time moving and more time encamped; (2) coyotes requiring more time to spend foraging and traveling to meet energetic demands in a prey-limited landscape (i.e., CBHNP) or (3) methodological differences (i.e., Ellington and Gehrt 2019 used data with a 15-min fix rate whereas our study used data with a 2 h fix rate).

Coyotes displayed changes in resource selection while foraging both across the diel cycle and seasonally that could indicate a shift in hunting or foraging strategy. Vision is the dominant sense used by coyotes for hunting (Wells 1978). Thus, during the day, open land cover with its longer sightlines would be advantageous to a visual predator, especially a cursorial predator like the coyote. At night, however, coyotes might rely more on auditory and olfactory cues for hunting because vision is reduced. During dawn and dusk, coyotes might also rely on increasing encounter rates with prey. We found evidence of this pattern during the snow-free season: foraging coyotes preferred open patches during the day but avoided open landscapes at night and instead selected for forest-open edges and heterogeneous landscapes. Coyotes and other cursorial predators are typically associated with edge habitats (Theberge and Wedeles 1989). It is thought that these transitions in land cover types offer higher prey availability because prey species that occur in two habitat types can occur in the same place. Moreover, foraging coyotes appear to select these landscapes when prey species (e.g., white-tailed deer and snowshoe hare) are most active (crepuscular period; Keith 1965; Beier and McCullough 1990).

Foraging coyotes altered their foraging strategy from the snow-free season to the snow season. In other systems, where there is deep snow coyotes tend to hunt and forage in forested land cover (Dowd et al. 2014), and our results support this finding at the landscape scale. During the snow season, foraging coyotes also preferred heterogenous landscapes and high elevation. The preference by coyotes for higher elevations during the snow season was unexpected—not only would foraging be more energetically costly at higher elevation (because of deeper snow), but presumably prey availability is lower at higher elevations because prey themselves are also subject to the higher energetic cost of moving in this landscape during the snow season. Yet, canids might have an advantage in deep snow. For example, wolves have higher ungulate kill rates in deep snow (Huggard 1993) and white-tailed deer are an important prey item for coyotes in Cape Breton (Patterson et al. 1998; Power et al. 2020). Furthermore, Patterson and Messier (2001) found that in the winter, coyotes in Cape Breton (south of the CBHNP) selected for areas with few to no deer and concluded that despite the decreased deer abundance in these areas (with deeper snow and few trails), deer vulnerability was higher. Finally, Patterson et al. (1998) found that snowshoe hare were the most common prey item of coyotes in Cape Breton during the winter and snowshoe hare are more common at higher elevations in CBHNP.

We found that coyotes responded to potential landscape risk across the diel cycle and seasonally in two ways: (1) by avoiding dangerous terrain when moving, especially in low light conditions; and (2) by dynamically responding to human features when human activity is most likely. We found that when moving (foraging and traveling), coyotes frequently avoided steep terrain, and that this response was consistent across behaviors during the night. During the day, however, this behavior was more acute when traveling than when foraging. This supports our prediction that coyotes will avoid potential landscape risk when traveling. This finding also indicates that the cost:benefit ratio of steep terrain when foraging might be perceived by coyotes as more costly in low light conditions. Interestingly, black-tailed deer (*Odocoileus hemionus*) in northern California avoid steep slopes when active but not when inactive; this is presumed to be an antipredator strategy (Bose et al. 2018). Yet, coyotes that were foraging during the day and during the snow season did not avoid steep slopes. This suggests that retreating to risky terrain for resting might not be an effective predator–avoidance strategy for the prey of coyotes in CBHNP all of the time.

Several studies have demonstrated that animals use temporal partitioning (Kronfeld-Schor and Dayan 2003) to balance the cost and benefits of occupying human dominated landscapes by temporally avoiding these areas when humans are more likely to be present or active (Wilson et al. 2012; Suraci et al. 2019; Zeller et al. 2019). We suggest that coyotes employ this temporal avoidance strategy to minimize risk due to human presence while maximizing benefits of human-dominated landscapes, namely prey hyperabundance (Fischer et al. 2012) and decreased energetic movement cost along trails and roads (Gese et al. 2013; Poessel et al. 2016). When foraging or traveling during the snow-free season, coyotes avoided areas with higher densities of linear corridors at the patch scale (traveling coyotes only avoided higher densities of linear corridors at the patch scale during the day) but selected for areas with higher densities of linear corridors at the landscape scale. This “close but not too close” strategy might allow coyotes to accrue the prey availability benefits of linear corridors without the costs of increased human presence. Similar to Wilson et al. (2012), we found that when traveling, coyotes avoided higher densities of linear corridors (at least when humans were presumably more active during the snow-free season). Conversely, during the snow season, foraging coyotes did not respond to linear corridor density at any scale, and traveling coyotes preferred higher linear corridor density at the patch scale. This suggests that coyotes did not associate any foraging benefit from linear corridors during the snow season, but did associate a traveling benefit. Indeed, researchers have repeatedly linked coyote movement in deep snow to linear corridors or other surfaces with packed snow that would reduce the energetic demand for coyotes (Crête and Larivière 2003). In CBHNP, human presence on these linear corridors is greatly reduced during the snow season.

We did not find any land cover associations with traveling coyotes during the snow season. Moving in snow is energetically costly for coyotes because they do not have morphological adaptations for moving in snow (e.g., the long limbs and low foot loads of lynx; Murray and Boutin 1991; Crête and Larivière 2003) and closed canopy forest and open landcover typically lead to very different underlying snow characteristics. Indeed, Pozzanghera et al. (2016) found that coyotes were associated with shallower, more compact snow, presumably associated with open land cover. Conversely, Droghini and Boutin (2018) found that a wolf’s ability to selectively travel in favorable snow conditions was limited in natural conditions but that they readily used roads and trails to reduce the energetic cost of moving in snow. It is possible that there is little variability in snow conditions across CBHNP in relation to landcover.

Although some carnivores, for example jaguars, appear to have consistent patterns of resource selection regardless of behavioral state (Gese et al. 2018), we found distinct responses in foraging and traveling behavior both across the diel cycle and seasonally for coyotes in CBHNP. Thus, our work supports the recommendations of Roever et al. (2014) and Abrahms et al. (2016) that oversimplification of resource selection analyses by ignoring behavioral state (and also diel and seasonal differences) can produce misleading results or cause important resource choices to be masked, ultimately reducing the effectiveness of management strategies. Indeed, while our analysis and discussion has largely focused on foraging and traveling behaviors, it is worth noting that we found that where coyotes chose to be encamped (presumably resting) was not related to any of the land cover, human disturbance, or terrain metrics we examined. Thus, this lack of choice by coyotes during approximately 1/3 of their monitoring would have masked the selection that was occurring for foraging and traveling coyotes had we not considered movement behavior. Of course, managers and researchers must balance increased specification of resource selection studies with increased data demands. For example, due to limited monitoring periods for individual coyotes (Appendix 2), we did not have sufficient sample size to robustly examine individual differences among coyotes in CBHNP. Yet, Zeller et al. (2019) found individual differences in resource choices in black bears even when accounting for behavioral state. Future studies might reveal that behaviorally explicit resource choices by coyotes are also individually variable, perhaps by a functional response to human presence or disturbance (as seen in wolves; Muhly et al. 2019), spatial learning (Merkle et al. 2019), or genotypic or phenotypic expressions (such as boldness; Wurth 2018).

## Electronic supplementary material

Below is the link to the electronic supplementary material.

Supplementary material 1 (PDF 4970 kb)
