# Supplementary material for: Seasonal and daily shifts in behavior and resource selection: how a carnivore navigates costly landscapes
Source: Oecologia. 2020 Sep 16;194(1):87–100. doi: 10.1007/s00442-020-04754-1 (PMC7561532; doi:10.1007/s00442-020-04754-1)
Supplement: Supplementary file 1 — Supplementary material 1 (PDF 4970 kb) [file 442_2020_4754_MOESM1_ESM.pdf]

Supplementary material for:

Seasonal and daily shifts in behavior and resource selection: how a carnivore navigates costly landscapes.

Journal: Oecologia

E. Hance Ellington[1,2], Erich M. Muntz[3], Stanley D. Gehrt[1]

[1] School of Environment and Natural Resources, Ohio State University, 210 Kottman Hall, 2021 Coffey Road, Columbus, OH 43210, USA

[2] present address: Range Cattle Research and Education Center, Wildlife Ecology and Conservation, University of Florida, 3401 Experiment Station Road, Ona, FL 33865, USA

[3] Cape Breton Highlands National Park, PO Box 158, Chéticamp, NS B0E1H0, Canada

e.hance.ellington@gmail.com

Appendix 1: Identifying coyote space use strategies

Identifying coyote space use strategies can be a challenging endeavor. Similar to Morin and Kelly (2017) and Sasmal et al. (2019), we suspected that coyotes (*Canis latrans*) in our study area of Cape Breton Highlands National Park (CBHNP) in Nova Scotia, Canada, employed at least three distinct strategies: residency, local transiency, and long-distance transiency. Coyotes often switch space use strategies over time. For example, long-distance and local transients might establish a territory and become a resident animal. A resident animal might leave its natal territory and become a local or long-distance transient. A resident animal might leave its territory after losing a mate or may even be displaced by another animal. A resident animal might leave one territory and begin occupying a different territory. A local transient might shift strategies and become a long-distance transient or vice versa.

First, we identified periods of consistent space use strategies within individual coyotes. Coyotes in our study were monitored using GPS collars and the fix rate of these collars varied among individuals and over time. To standardize our analysis, we rarified our dataset to 7 hr (the longest fix rate present in the dataset) by generating trajectories using adehabitatLT (Calenge 2006) in R (R Core Team 2020). We then extracted the net-squared displacement from each animal's trajectory. We plotted net-squared displacement over time to visualize patterns in space use. Within these plots we saw three distinct patterns but often we saw multiple patterns within a single individual (7 of 16 individuals, e.g., Figure 1d). When we saw multiple patterns within a single individual, we then manually investigated the locations of that individual in ArcGIS (ESRI 2020). Here we used series of two-week location data to estimate the two-week interval when that animal changed its space use pattern (Figure 2). Once we had narrowed the transition down to a two-week period, we then manually moved chronologically through the locations within that

two-week period to estimate a precise time that the animal transitioned from one space use pattern to another. This process produced 20 bursts of consistent space use and 8 bursts with insufficient data from 16 animals (hereafter, individual-burst).

Next, we classified individual-bursts as distinct space use strategies (residency, local transiency, or long-distance transiency). To estimate space use strategy, we used a combination of information:

1) Patterns in net-squared displacement over time
2) Field observations from Jason Power and CBHNP staff (Table 1 in Appendix 2)
3) Spatial pattern in location data both within the individual-burst of location data and among other individual-bursts of location data that overlapped temporally.

When we plotted net-squared displacement over time for individual-bursts, three general patterns observed: 1) a single band of NSD, perhaps with a few brief spikes of NSD that is indicative of a resident animal (Figure 1a); 2) a single band of NSD but with frequent large spikes of NSD that is indicative of a local transient (Figure 1b); or 3) an arc or trajectory of NSD values, rather than a band, and in general NSD values that were 1-2 orders of magnitude greater than those observed in the first two patterns, indicative of a long-distance transient animal (Figure 1c). It should be noted that for individual-bursts that were less than 60 days, it was often difficult to discern a consistent pattern in NSD (we either perceived a series of transitions between different space use patterns or could not easily determine the difference between pattern #1 and pattern #2 (Figure 3). We then compared field observations that corresponded to individual-bursts. In most cases, when field observations indicated residency (animal with signs of estrous, found den site, or observed with pups) it also corresponded to pattern #1. There was one case in which an animal was observed with pups but the NSD pattern matched pattern #2 in this case we classified this

animal as a local transient. There was also one case in which an animal that was monitored for less than 60 days was also observed with pups but given the limited spatial data we had available we classified this animal as an unknown space use strategy. Our final tool was to manually inspect spatial pattern of individual-burst location data – here we were looking for:

1) Regularly reused areas (suggestive of residency) and that these suggestive residents did not use areas that overlapped with other suggestive residents (in the same time period; unless they completely overlapped, suggesting members of the same family group). This behavior corresponded to NSD pattern #1.
2) Reused areas but made frequent bouts of movement into other reused areas (some of these areas might overlap with other individuals in the same time period, but overlap was only partial and was often restricted to only one of the areas these animals regularly reused). This behavior corresponded to NSD pattern #2.
3) Did not reuse areas for more than a few weeks and travelled long-distances across the landscape. This behavior corresponded to NSD pattern #3.

Thus, we linked observed patterns in NSD data to distinct space use patterns that corresponded to our trinary classification of space use strategy. Moreover, in most cases these NSD and space use patterns corresponded to available field observations related to residency. We had only one case of potential disagreement – coyote 23 in which field observations indicated residency but NSD and space use patterns indicated local transiency. Here it is worth noting that there are many facets of coyote behavior that are still unknown. For example, we know that the alpha pair will occasionally tolerate adult (or subadult) family members to reside within the territory but in other cases, these individuals are forced out of the territory when new pups are born. However, we don't know how ardently alphas defend their territories against occasional incursions by highly

related individuals. So, it is possible that a related individual that mostly lives outside of the territory as a local transient is tolerated and is occasionally moving with pups or alphas.

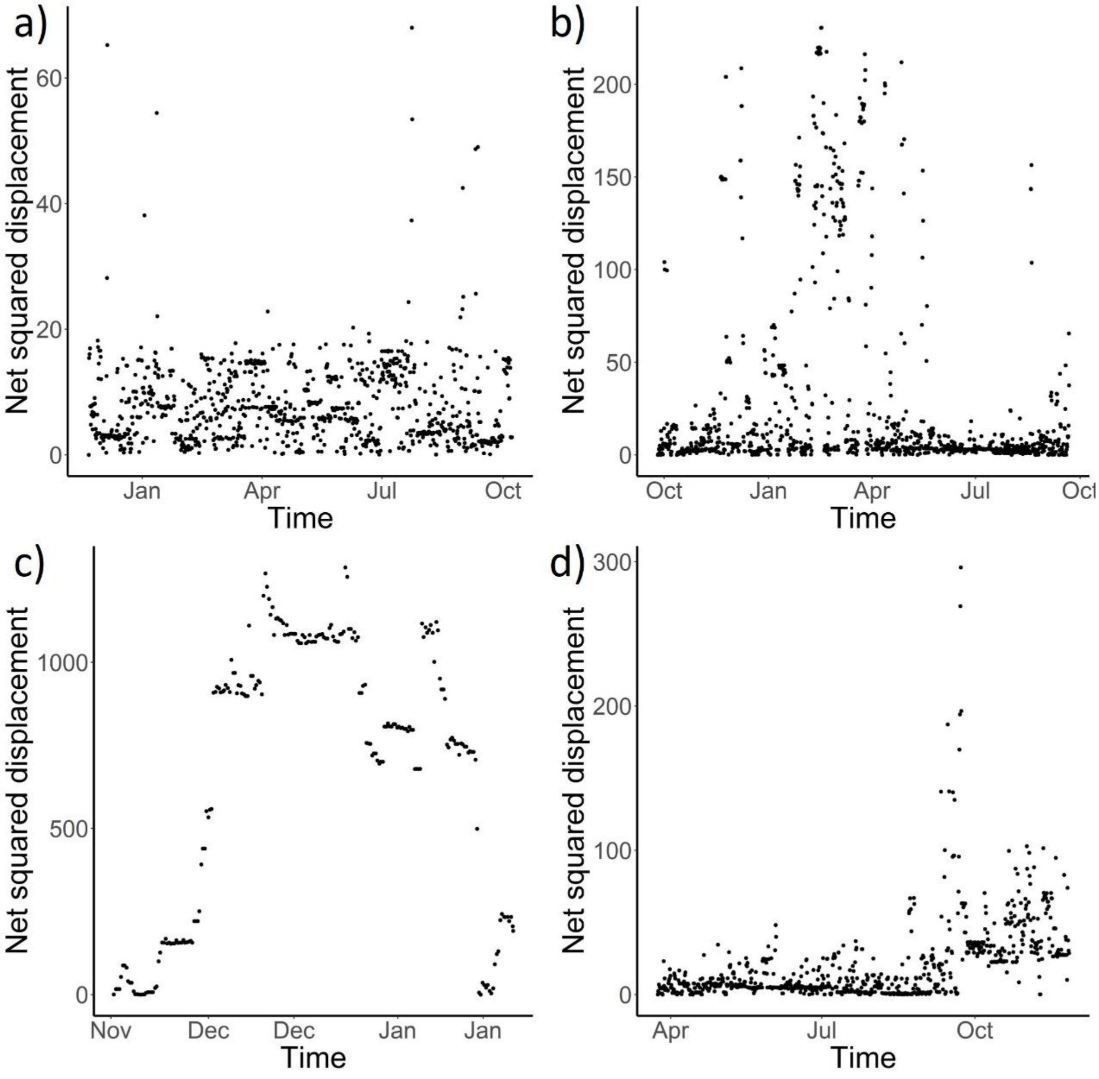

Figure 1. Net-squared displacement (NSD) of trajectories of coyotes (*Canis latrans*) monitored using GPS collars rarified to a 7hr fix rate over time from October 2011 to September 2016 in Cape Breton Highlands National Park, Nova Scotia, Canada. We saw three distinct patterns in these NSD plots: a single band of NSD perhaps with occasional short spikes in NSD (a), a single band of NSD with frequent large spikes in NSD (b), an arc of NSD that is often orders of magnitude larger than that observed in other patterns of NSD (c). Within individuals we occasionally observed multiple patterns of NSD (d) that required further investigation to delineate into multiple bursts of unique NSD patterns within an individual.

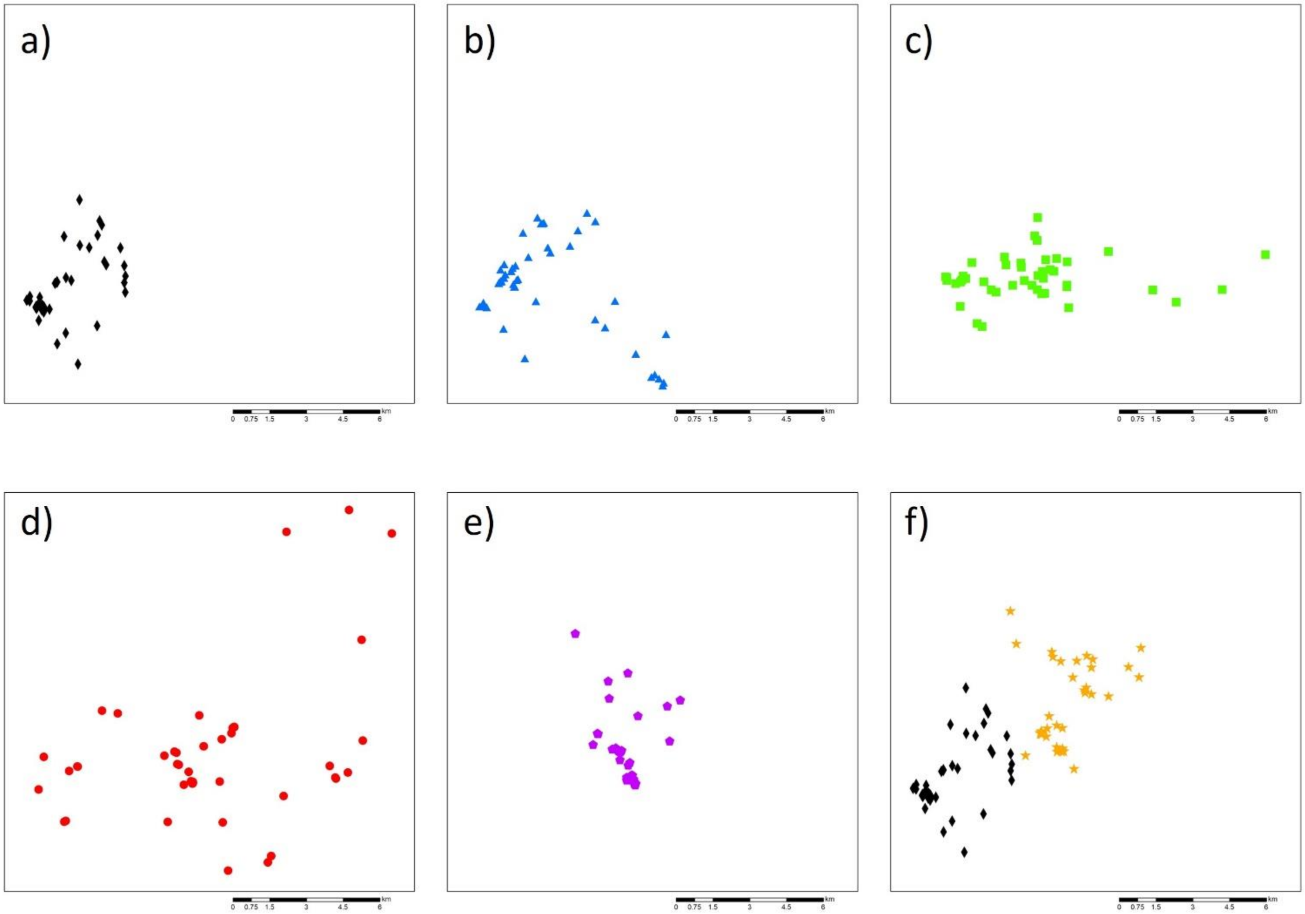

Figure 2. Space use patterns of an individual coyote (*Canis latrans*) in two-week intervals (a-e) showing the transition from one space use strategy to another space use strategy. The black diamonds (a) and blue triangles (b) show the animal employing space use strategy #1 and the green squares (c) and red circles (d) show the animal during the transition to a different space use strategy. The purple hexagons (e) and the orange stars (f) show the animal employing space use strategy #2. This animal was monitored using a GPS collar with fixes rarified to a 7hr fix rate in Cape Breton Highlands National Park, Nova Scotia, Canada.

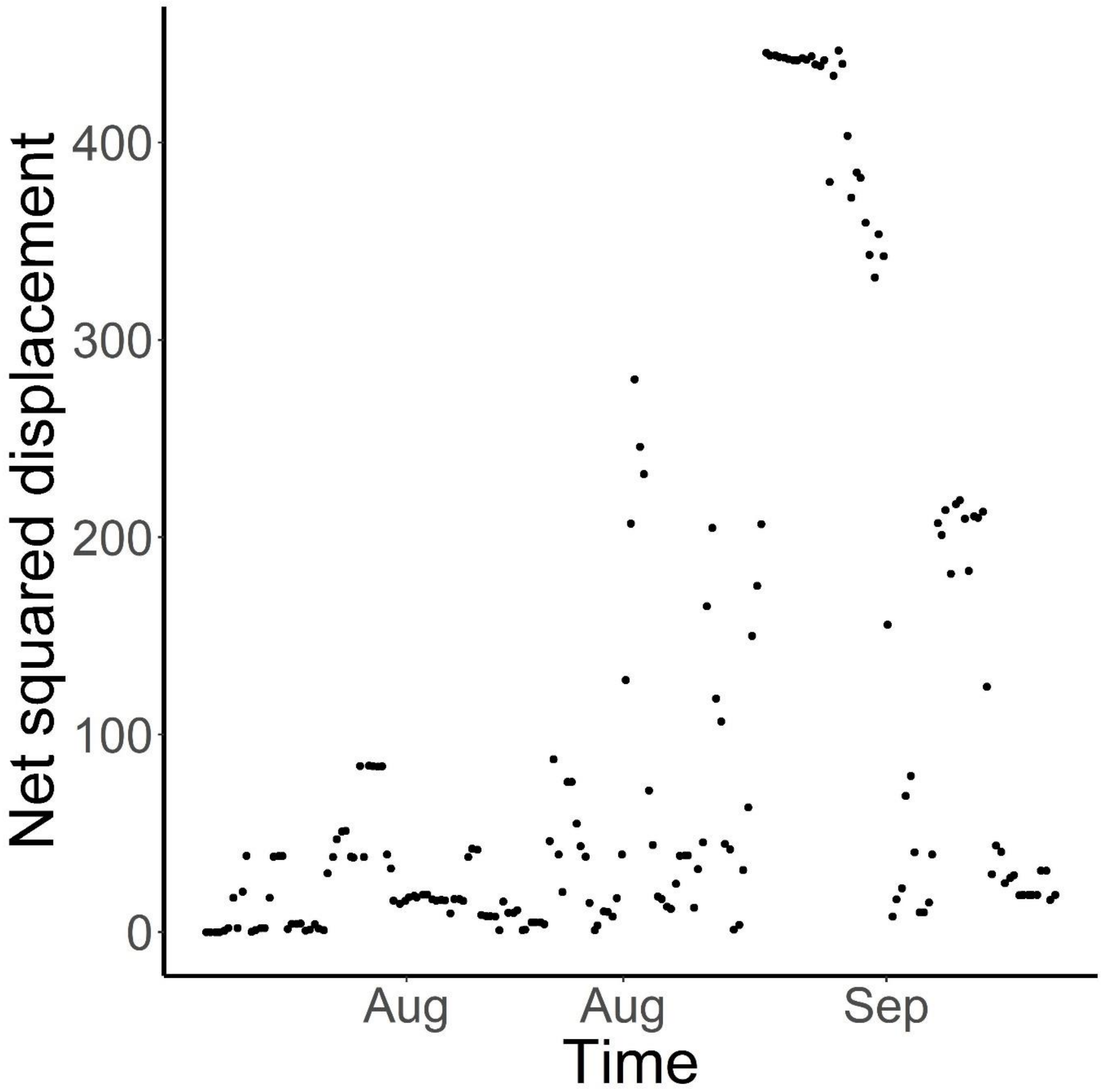

Figure 3. Net-squared displacement (NSD) of a trajectory of coyotes (*Canis latrans*) monitored using GPS collars rarified to a 7hr fix rate over time in Cape Breton Highlands National Park, Nova Scotia, Canada. This individual was monitored for less than 60 days which made it difficult to estimate a consistent pattern in NSD. For example, we could either classify this individual as displaying NSD pattern #2 (see Figure 1b) or displaying multiple NSD patterns with a transition occurring in mid-August (see Figure 1).

Literature Cited

Calenge C (2006). The package “adehabitat” for the R software: A tool for the analysis of space and habitat use by animals. Ecol Model 197:516–519. https://doi.org/10.1016/j.ecolmodel.2006.03.017

ESRI (2020). ArcGIS Desktop: Release 10.8. Redlands, CA: Environmental Systems Research Institute.

Morin DJ, Kelly MJ (2017). The dynamic nature of territoriality, transience and biding in an exploited coyote population. Wildl Biol 2017:

R Core Team (2020). R: A language and environment for statistical computing. R Foundation for Statistical Computing, Vienna, Austria.

Sasmal I, Moorman CE, Swingen MB, Datta S, DePerno, CS (2019). Seasonal space use of transient and resident coyotes (*Canis latrans*) in North Carolina, USA. Can J Zool 97:326–331. https://doi.org/10.1139/cjz-2018-0209

Appendix 2. Summary statistics and maps of individual coyote space use and movement

In this appendix we report the monitoring period, field observations, and home range size for individual-bursts (periods of unique space use strategy for each individual) of coyotes (*Canis latrans*) monitored using GPS collars from October 2011 to September 2016 in Cape Breton Highlands National Park, Nova Scotia, Canada (Table 1). We also report summary data on seasonal home ranges when available (Table 2). We report the timing and missing location rate of individual bursts of 2-hour fix rate data that we used to estimate coyote movement behavior using Hidden Markov Models (Table 3). Finally, we visualize resident home ranges, local transient home ranges, and location data of long-distance transients on an aerial image of our study area (Figures 1-20).

Table 1. Summary of home range size, space use strategy (if known), and sex of coyotes (*Canis latrans*), monitored using GPS collars from October 2011 to September 2016 in Cape Breton Highlands National Park, Nova Scotia, Canada.

Coyote ID[a]	Sex	Notes on social status[b]	First location	Last location	Number of days monitored	95% LCH	95% MCP
Resident							
1a	M		10/28/2011 23:02	2/11/2012 11:01	106	70.6	109.1
1b	M		2/11/2012 18:00	6/19/2012 10:01	129	28.9	36.4
3a	M		1/17/2012 9:01	7/31/2012 8:01	196	23.3[c]	34.1
5b	M	Found with two other coyotes	1/8/2012 2:00	4/3/2012 18:00	87	27.9	57.3
5d	M	Found with two other coyotes	4/24/2012 18:00	7/14/2012 17:00	81	22.8	40.4
6	M	Tracked with five other coyotes	11/21/2011 0:01	10/7/2012 13:31	322	29.0	31.2
7	M		6/27/2012 23:00	9/17/2012 5:01	81	7.5	12.5
8b	M		9/30/2012 9:40	6/15/2013 11:06	258	19.6	25.0
11	F	Tracked with three other coyotes; signs of estrous	10/15/2012 21:01	3/26/2013 8:00	161	36.0	74.7
18b	M	Found with estrous female	2/3/2013 17:00	4/16/2013 13:01	72	7.7	14.0
19	M	Found suspected den site	6/7/2013 3:01	12/9/2013 9:00	185	36.4	101.7
26	M		11/17/2013 2:01	9/19/2014 7:00	306	23.9	27.0
1501c	F		7/9/2015 17:01	9/14/2015 7:00	67	10.8	18.8
1502a	M		3/23/2015 21:00	9/15/2015 13:00	176	26.7	34.6
Local transient							
3b	M		10/31/2011 21:01	1/17/2012 2:01	77	80.8	212.8
23	M	Seen with pups	7/15/2013 1:01	2/7/2014 15:01	208	113.9	370.7
1501b	F		3/28/2015 20:00	7/9/2015 11:00	103	98.1	221.1
1502b	M		9/15/2015 19:00	11/26/2015 12:45	72	44.6	72.4
1503	M		9/25/2015 3:00	9/21/2016 4:00	362	82.0	153.8
Long-distance transient							
25	M		11/15/2013 2:00	1/19/2014 18:00	66		

			Insufficient data		
5a	M		11/14/2011 0:01	1/7/2012 19:01	55
5c	M		4/4/2012 1:01	4/24/2012 11:01	20
8a	M		7/18/2012 15:00	9/11/2012 19:00	55
13	F	Found den site with three pups	11/15/2012 12:02	12/12/2012 10:02	27
18a	M		11/12/2012 0:01	12/7/2012 16:00	26
18c	M		7/26/2013 23:00	8/2/2013 9:51	6
21	M		6/26/2013 9:03	7/26/2013 15:01	30
1501a	F		3/20/2015 2:00	3/28/2015 16:00	9

[a] Coyote ID contains information both on the unique individual (number) and unique space use strategies employed by that animal during monitoring (letter). Additionally, note that some individuals were residents in two distinct home ranges.

[b] Notes on social status were derived from field notes based on snow-tracking collared coyotes by Jason Power and CBHNP staff.

[c] The 95% isopleth of the adaptive local convex hull polygon was derived using the 99.5% percentile of the maximum distance in the distance matrix of all fixes. This estimate is likely an underestimate relative to the other home range size estimates.

Table 2. Summary of seasonal home range size of coyotes (*Canis latrans*) monitored using GPS collars from October 2011 to September 2016 in Cape Breton Highlands National Park, Nova Scotia, Canada.

Coyote ID[a]	No Snow[b]					Snow[b]				
	First location	Last location	Number of days monitored	95% LCH	95% MCP	First location	Last location	Number of days monitored	95% LCH	95% MCP
Resident										
1a						11/22/2011 4:00	2/11/2012 11:01	81	69.7	126.8
1b	4/15/2012 2:01	6/19/2012 10:01	65	22.5	33.2	2/11/2012 18:00	4/14/2012 19:02	63	23.4	27.1
3a	4/15/2012 2:00	7/31/2012 8:01	107	17.9[c]	24.2	1/17/2012 9:01	4/14/2012 19:00	88	22.5	38.1
5b						1/8/2012 2:00	4/3/2012 18:00	87	27.9	57.3
5d	4/24/2012 18:00	7/14/2012 17:00	81	22.8	40.4					
6	4/15/2012 3:00	10/7/2012 13:31	175	28.1	30.7	11/22/2011 4:01	4/14/2012 20:00	145	21.1[c]	27.6
7	6/27/2012 23:00	9/17/2012 5:01	81	7.5	12.5					
8b	4/16/2013 5:01	6/15/2013 11:06	60	5.5	7.3	11/21/2012 1:00	4/15/2013 22:01	146	10.3	15.3
11						11/21/2012 0:01	3/26/2013 8:00	125	39.6	75.3
18b						2/3/2013 17:00	4/15/2013 23:02	71	7.5	14.0
19	6/7/2013 3:01	11/21/2013 21:01	168	30.1	36.5					
26	4/16/2014 1:01	9/19/2014 7:00	156	43.5[c]	34.6	11/22/2013 2:00	4/15/2014 19:00	145	13.5	20.6

1501c	7/9/2015 17:01	9/14/2015 7:00	67	10.8	18.8					
1502a	4/16/2015 5:01	9/15/2015 13:00	152	24.1	31.6					
					Local transient					
23	7/15/2013 1:01	11/21/2013 16:03	130	41.4	162.4	11/22/2013 6:01	2/7/2014 15:01	77	119.4	296.2
1501b	4/16/2015 6:01	7/9/2015 11:00	84	92.6	216.7					
1502b	9/15/2015 19:00	11/21/2015 20:00	67	45.4	72.9					
1503	4/15/2016 1:00	9/21/2016 4:00	159	29.5	50.3	11/22/2015 2:00	4/14/2016 19:00	145	71.4	153.7

[a] Coyote ID contains information both on the unique individual (number) and unique space use strategies employed by that animal during monitoring (letter). Additionally, note that some individuals were residents in two distinct home ranges.

[b] The snow-free season was estimated as April 14th – 15th – November 20th – 21st and the snow season was estimated as April 15th - 16th – November 21st – 22nd.

[c] The 95% isopleth of the adaptive local convex hull polygon was derived using the 99.5% percentile of the maximum distance in the distance matrix of all fixes. This estimate is likely an underestimate relative to the other home range size estimates.

Table 3. Summary of timing and missing rate of locations for movement bursts used to estimate movement behavior of coyotes (*Canis latrans*) monitored with GPS collars recording locations every 2 hours from October 2011 to October 2015 in Cape Breton Highlands National Park, Nova Scotia, Canada.

ID	Unique burst	Number of locations	First location	Last location	Days monitored	Missing rate
1	1.2	145	11/05/2011 19:00	11/18/2011 14:01	76.9	0.03
1	1.3	148	11/19/2011 10:00	12/01/2011 22:01	61.2	0.08
3	3.01	139	10/31/2011 23:00	11/14/2011 04:01	53.2	0.07
3	3.02	193	11/14/2011 14:01	12/01/2011 20:01	40.8	0.11
3	3.08	128	06/23/2012 03:00	07/05/2012 17:00	29.2	0.02
5	5.1	143	11/13/2011 22:00	11/26/2011 00:01	29.1	0.09
5	5.3	215	12/01/2011 14:00	12/20/2011 22:00	26.7	0.03
5	5.4	292	06/01/2012 03:01	06/27/2012 15:00	29.7	0.13
5	5.5	103	06/28/2012 05:02	07/08/2012 17:01	26.5	0.08
6	6.2	342	11/21/2011 18:01	12/20/2011 22:00	27.3	0.12
6	6.3	204	06/01/2012 05:00	06/19/2012 11:00	23.4	0.11
6	6.4	112	06/20/2012 01:00	06/29/2012 19:01	23.4	0.12
6	6.6	221	07/04/2012 21:01	07/26/2012 09:00	21.3	0.08
7	7.01	172	06/28/2012 01:00	07/14/2012 01:01	21.5	0.14
7	7.05	204	07/18/2012 01:00	08/06/2012 23:01	20.2	0.09
7	7.08	115	08/14/2012 21:01	08/26/2012 01:02	19.3	0.07
8	8.1	130	07/18/2012 13:00	07/30/2012 19:00	18.6	0.06
8	8.3	435	08/01/2012 23:01	09/11/2012 17:01	17.8	0.02
11	11.1	897	10/15/2012 23:00	12/31/2012 20:00	19.9	0.15
13	13.1	248	11/15/2012 14:01	12/09/2012 00:01	18.3	0.07
18	18.1	119	11/11/2012 22:00	11/22/2012 06:00	17.3	0.07
19	19.2	220	06/10/2013 19:01	07/01/2013 01:00	16.3	0.09
19	19.3	249	07/01/2013 11:02	07/24/2013 21:01	16.9	0.14
23	23.07	174	08/03/2013 21:01	08/20/2013 19:00	14.8	0.02
25	25.1	593	11/14/2013 22:00	01/07/2014 02:00	16	0.1
25	25.2	133	01/07/2014 12:00	01/19/2014 16:01	14.8	0.06
26	26.01	209	11/17/2013 00:01	12/05/2013 14:00	16.7	0.2
26	26.02	166	12/06/2013 00:00	12/20/2013 18:01	14.6	0.11
26	26.03	209	12/21/2013 04:00	01/08/2014 00:00	12.5	0.01
26	26.04	235	01/08/2014 12:00	01/29/2014 20:00	12.8	0.05
26	26.07	311	02/14/2014 12:00	03/13/2014 05:00	12.8	0.06
26	26.08	672	03/13/2014 15:00	05/13/2014 19:00	12.1	0.01
26	26.11	133	05/24/2014 05:00	06/05/2014 23:00	13.2	0.12
26	26.14	178	06/15/2014 19:00	07/02/2014 01:00	12.8	0.13
26	26.2	159	07/16/2014 13:01	08/02/2014 05:01	12.2	0.09

26	26.31	174	09/04/2014 09:00	09/19/2014 05:00	12.3	0.12
1502	1502.01	309	03/23/2015 23:01	04/22/2015 15:01	12.6	0.15
1502	1502.02	316	04/23/2015 03:00	05/22/2015 05:00	10.3	0.04
1502	1502.04	155	08/15/2015 01:00	08/29/2015 15:00	11.7	0.16
1502	1502.06	146	08/30/2015 23:01	09/12/2015 19:00	11.2	0.14
1502	1502.07	290	09/13/2015 05:00	10/10/2015 13:00	9.8	0.04
1503	1503.06	118	10/17/2015 19:00	10/29/2015 11:00	10.5	0.18

Figure 1. Home range of coyote 1, a resident male from 28 October 2011 – 11 February 2012 (dashed line) and snow season home range from 22 November 2011 – 11 February 2012 (blue) in Cape Breton Highlands National Park, Nova Scotia, Canada. Home ranges were estimated using the 95% isopleth of the adapted local convex hull polygon method from GPS telemetry data collected every 7 hours.

Figure 2. Home range of coyote 1, a resident male from 11 February 2012 – 19 June 2012 (dashed line), snow-free season home range from 15 April 2012 – 19 June 2012 (red), and snow season home range from 11 February 2012 – 14 April 2012 (blue) in Cape Breton Highlands National Park, Nova Scotia, Canada. Home ranges were estimated using the 95% isopleth of the adaptive local convex hull polygon method from GPS telemetry data collected every 7 hours.

Figure 3. Home range of coyote 3, a resident male from 17 January 2012 – 31 July 2012 (dashed line), snow-free season home range from 15 April 2012 – 31 July 2012 (red), and snow season home range from 17 January 2012 – 14 April 2012 (blue) in Cape Breton Highlands National Park, Nova Scotia, Canada. Home ranges were estimated using the 95% isopleth of the adapted local convex hull polygon method from GPS telemetry data collected every 7 hours. Adaptive local convex hull polygons were derived using the 99.5% percentile of the maximum distance in the distance matrix of all fixes

Figure 4. Home range of coyote 5, a resident male from 8 January 2012 – 3 April 2012 (dashed line), which fell entirely during the snow season in Cape Breton Highlands National Park, Nova

Scotia, Canada. Home ranges were estimated using the 95% isopleth of the adapted local convex hull polygon method from GPS telemetry data collected every 7 hours.

Figure 5. Home range of coyote 5, a resident male from 24 April 2012 – 14 July 2012 (dashed line), which fell entirely during the snow-free season in Cape Breton Highlands National Park, Nova Scotia, Canada. Home ranges were estimated using the 95% isopleth of the adapted local convex hull polygon method from GPS telemetry data collected every 7 hours.

Figure 6. Home range of coyote 6, a resident male from 21 November 2011 – 7 October 2012 (dashed line), snow-free season home range from 15 April 2012 – 7 October 2012 (red), and snow season home range from 22 November 2011 – 14 April 2012 (blue) in Cape Breton Highlands National Park, Nova Scotia, Canada. Home ranges were estimated using the 95% isopleth of the adapted local convex hull polygon method from GPS telemetry data collected every 7 hours. Adaptive local convex hull polygons were derived using the 99.5% percentile of the maximum distance in the distance matrix of all fixes

Figure 7. Home range of coyote 7, a resident male from 27 June 2012 – 17 September 2012 (dashed line), which fell entirely during the snow-free season in Cape Breton Highlands National Park, Nova Scotia, Canada. Home ranges were estimated using the 95% isopleth of the adapted local convex hull polygon method from GPS telemetry data collected every 7 hours.

Figure 8. Home range of coyote 8, a resident male from 30 September 2012 – 15 June 2013 (dashed line), snow-free season home range from 16 April 2013 – 15 June 2013 (red), and snow

season home range from 21 November 2012 – 15 April 2013 (blue) in Cape Breton Highlands National Park, Nova Scotia, Canada. Home ranges were estimated using the 95% isopleth of the adapted local convex hull polygon method from GPS telemetry data collected every 7 hours.

Figure 9. Home range of coyote 11, a resident female from 15 October 2012 – 26 March 2013 (dashed line) and snow season home range from 21 November 2012 – 26 March 2013 (blue) in Cape Breton Highlands National Park, Nova Scotia, Canada. Home ranges were estimated using the 95% isopleth of the adapted local convex hull polygon method from GPS telemetry data collected every 7 hours.

Figure 10. Home range of coyote 18, a resident male from 3 February 2013 – 16 April 2013 (dashed line) and snow season home range from 3 February 2013 – 15 April 2013 (blue) in Cape Breton Highlands National Park, Nova Scotia, Canada. Home ranges were estimated using the 95% isopleth of the adapted local convex hull polygon method from GPS telemetry data collected every 7 hours.

Figure 11. Home range of coyote 19, a resident male from 7 June 2013 – 9 December 2013 (dashed line) and snow-free season home range from 7 June 2013 – 21 November 2013 (red) in Cape Breton Highlands National Park, Nova Scotia, Canada. Home ranges were estimated using the 95% isopleth of the adapted local convex hull polygon method from GPS telemetry data collected every 7 hours.

Figure 12. Home range of coyote 26, a resident male from 17 November 2013 – 19 September 2014 (dashed line), snow-free season home range from 16 April 2014 – 19 September 2014 (red), and snow season home range from 22 November 2013 – 15 April 2014 (blue) in Cape Breton Highlands National Park, Nova Scotia, Canada. Home ranges were estimated using the 95% isopleth of the adapted local convex hull polygon method from GPS telemetry data collected every 7 hours. Adaptive local convex hull polygons were derived using the 99.5% percentile of the maximum distance in the distance matrix of all fixes

Figure 13. Home range of coyote 1501, a resident female from 9 July 2015 – 14 September 2015 (dashed line), which fell entirely within the snow-free season in Cape Breton Highlands National Park, Nova Scotia, Canada. Home ranges were estimated using the 95% isopleth of the adapted local convex hull polygon method from GPS telemetry data collected every 7 hours.

Figure 14. Home range of coyote 1502, a resident male from 23 March 2015 – 15 September 2015 (dashed line) and snow-free season home range from 16 April 2015 – 15 September 2015 (red) in Cape Breton Highlands National Park, Nova Scotia, Canada. Home ranges were estimated using the 95% isopleth of the adapted local convex hull polygon method from GPS telemetry data collected every 7 hours.

Figure 15. Home range of coyote 3, a local transient male from 31 October 2011 – 17 January 2012 (dashed line) in Cape Breton Highlands National Park, Nova Scotia, Canada. Home ranges were estimated using the 95% isopleth of the adapted local convex hull polygon method from GPS telemetry data collected every 7 hours.

Figure 16. Home range of coyote 23, a local transient male from 15 July 2013 – 7 February 2014 (dashed line), snow-free season home range from 15 July 2013 – 21 November 2013 (red), and snow season home range from 22 November 2013 – 7 February 2014 (blue) in Cape Breton Highlands National Park, Nova Scotia, Canada. Home ranges were estimated using the 95% isopleth of the adapted local convex hull polygon method from GPS telemetry data collected every 7 hours.

Figure 17. Home range of coyote 1501, a local transient female from 28 March 2015 – 9 July 2015 (dashed line) and snow-free season home range from 16 April 2015 – 9 July 2015 (red) in Cape Breton Highlands National Park, Nova Scotia, Canada. Home ranges were estimated using the 95% isopleth of the adapted local convex hull polygon method from GPS telemetry data collected every 7 hours.

Figure 18. Home range of coyote 1502, a local transient male from 15 September 2015 – 26 November 2015 (dashed line) and snow-free season home range from 15 September 2015 – 21 November 2015 (red) in Cape Breton Highlands National Park, Nova Scotia, Canada. Home ranges were estimated using the 95% isopleth of the adapted local convex hull polygon method from GPS telemetry data collected every 7 hours.

Figure 19. Home range of coyote 1503, a local transient male from 25 September 2015 – 21 September 2016 (dashed line), snow-free season home range from 15 April 2016 – 21 September 2016 (red), and snow season home range from 22 November 2015 – 14 April 2016 (blue) in Cape Breton Highlands National Park, Nova Scotia, Canada. Home ranges were estimated using

the 95% isopleth of the adapted local convex hull polygon method from GPS telemetry data collected every 7 hours.

Figure 20. Locations of coyote 25, a long-distance transient male from 15 November 2013 – 19 January 2014 in Cape Breton Highlands National Park, Nova Scotia, Canada.

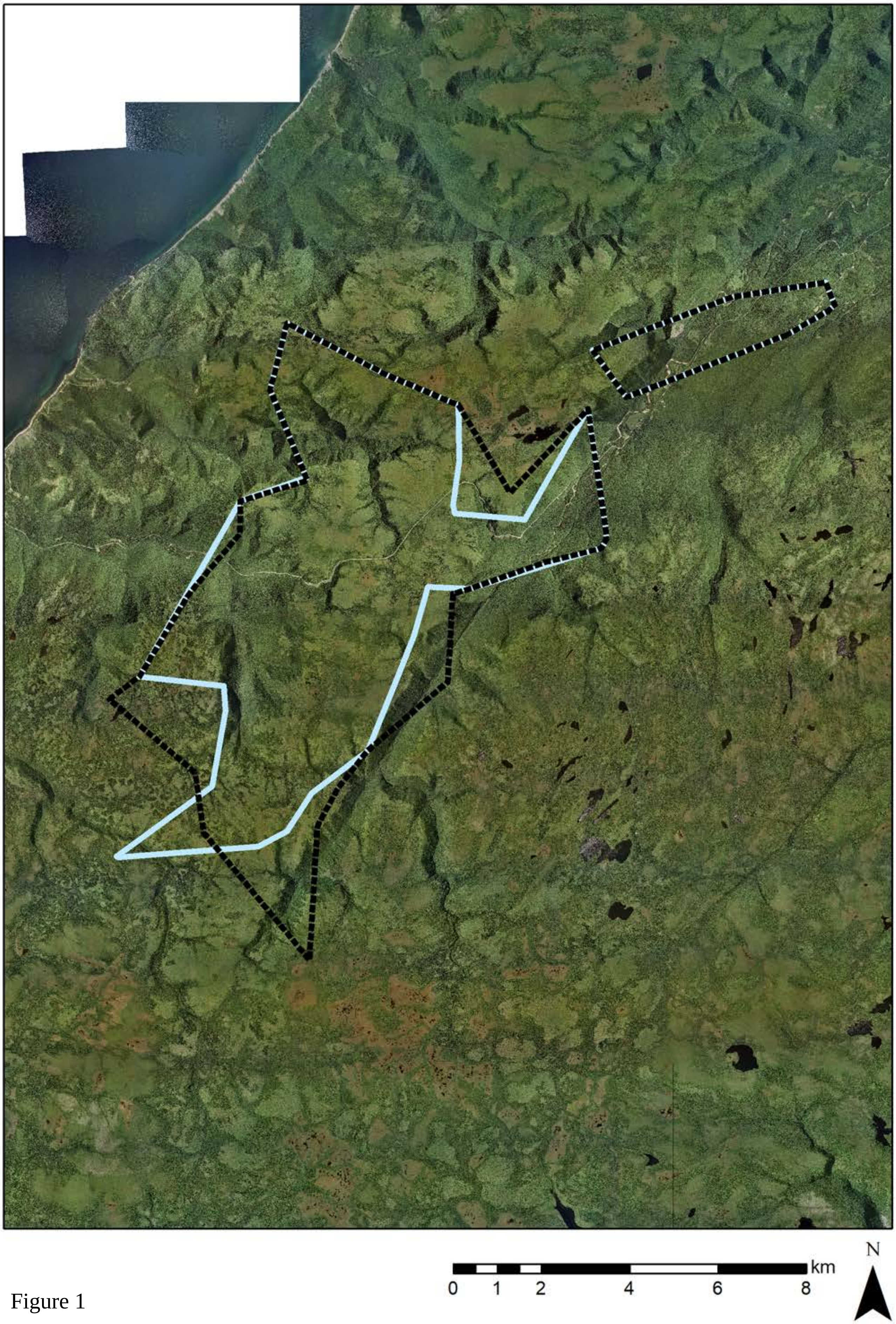

Figure 1

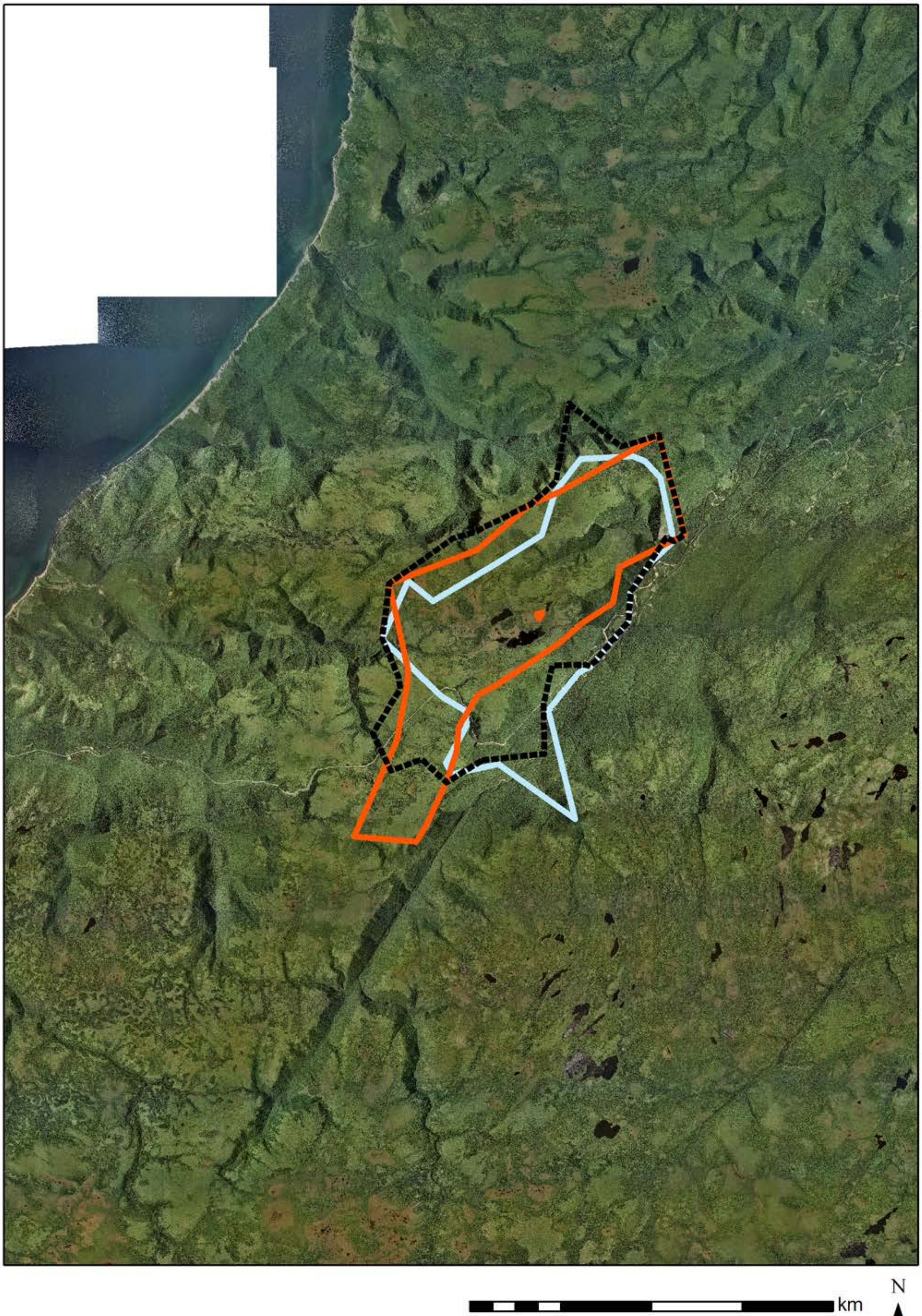

Figure 2

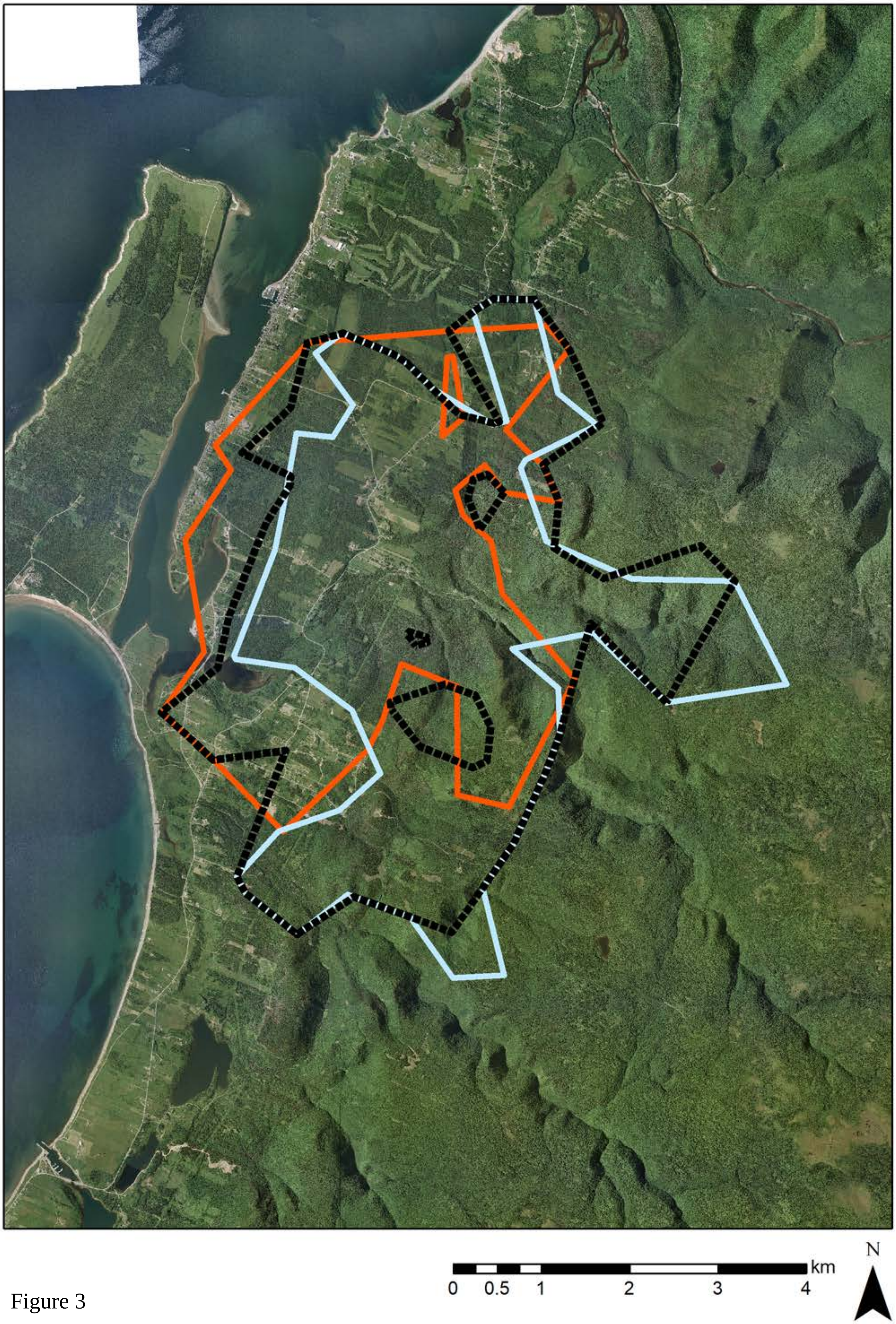

Figure 3

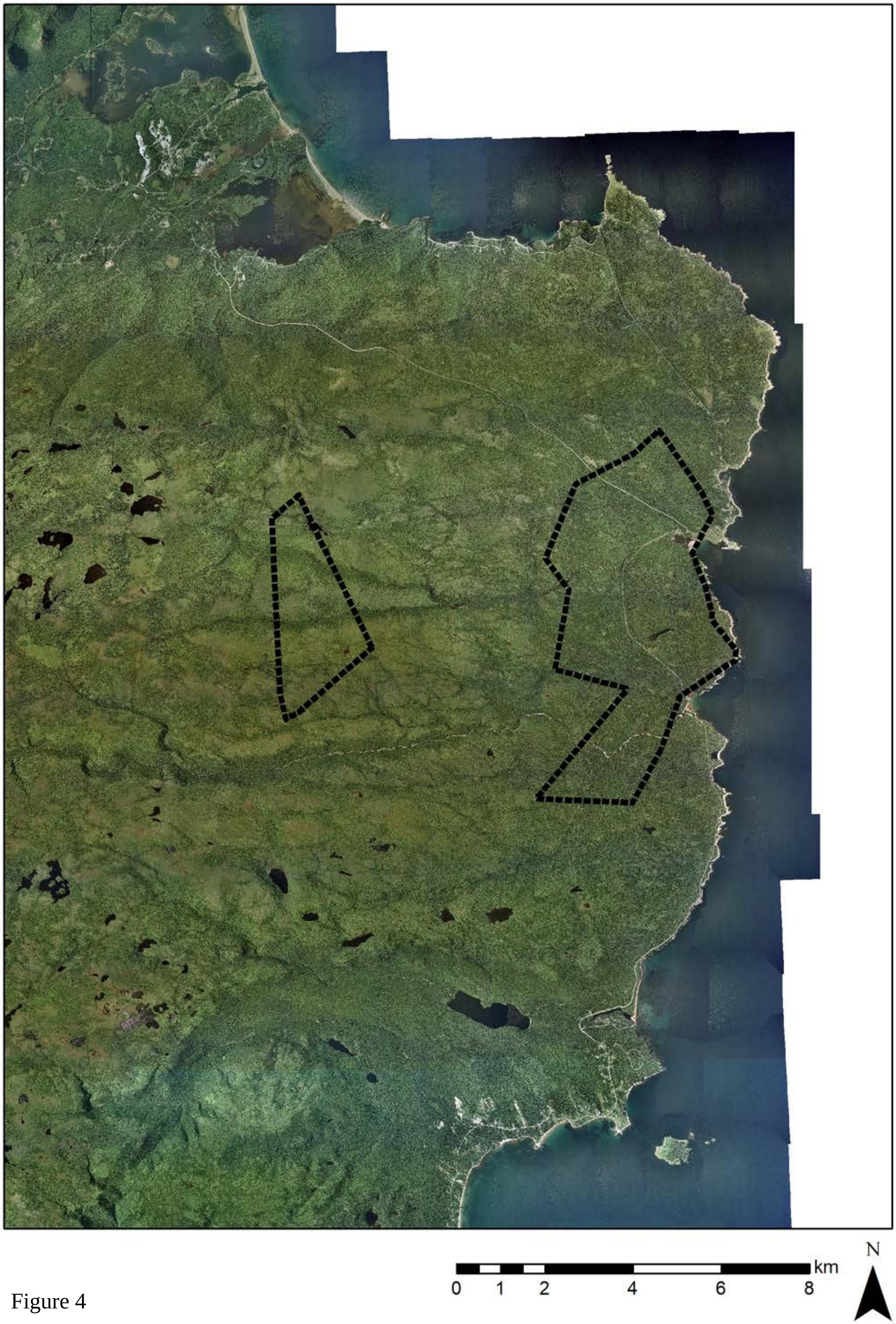

Figure 4

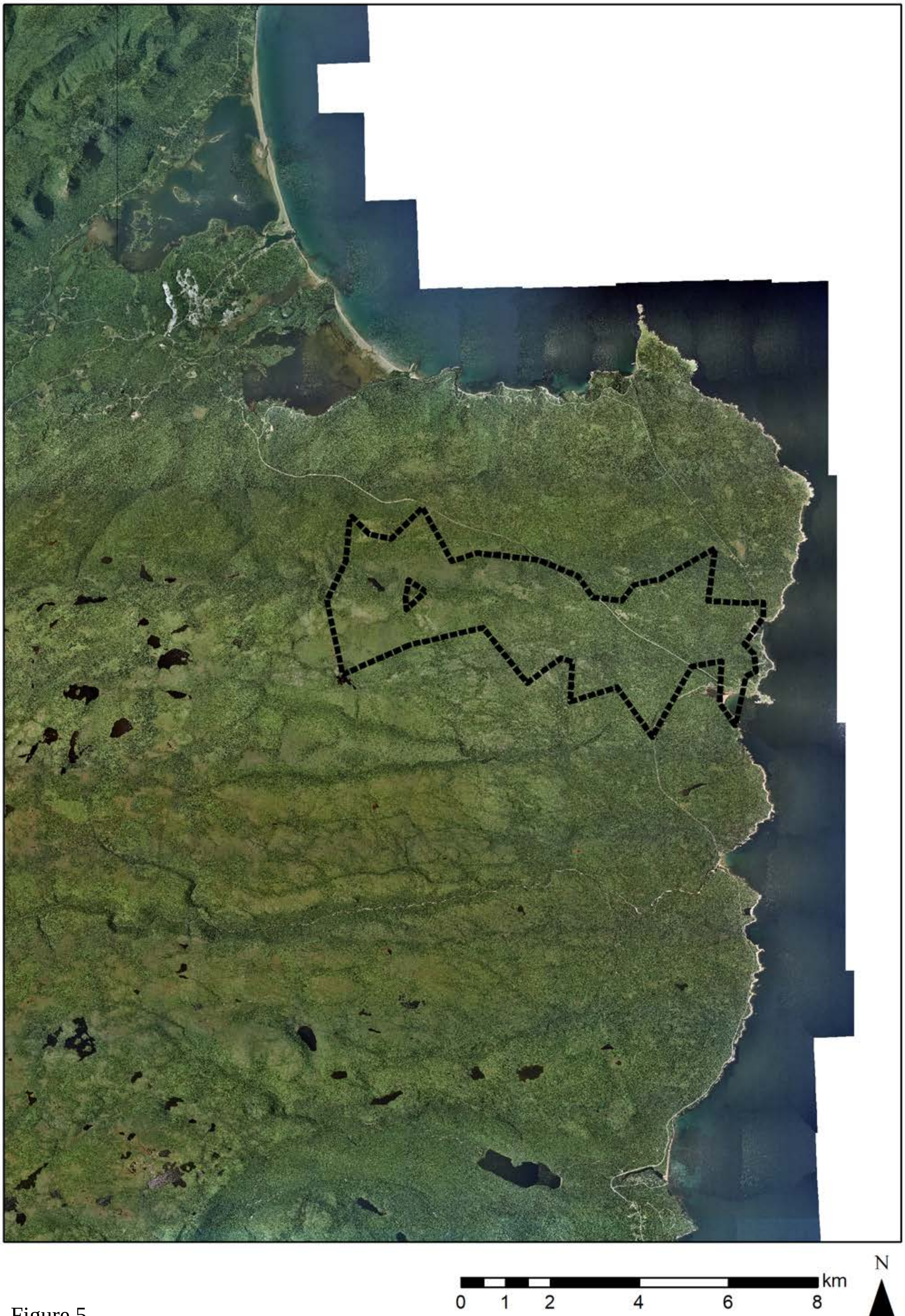

Figure 5

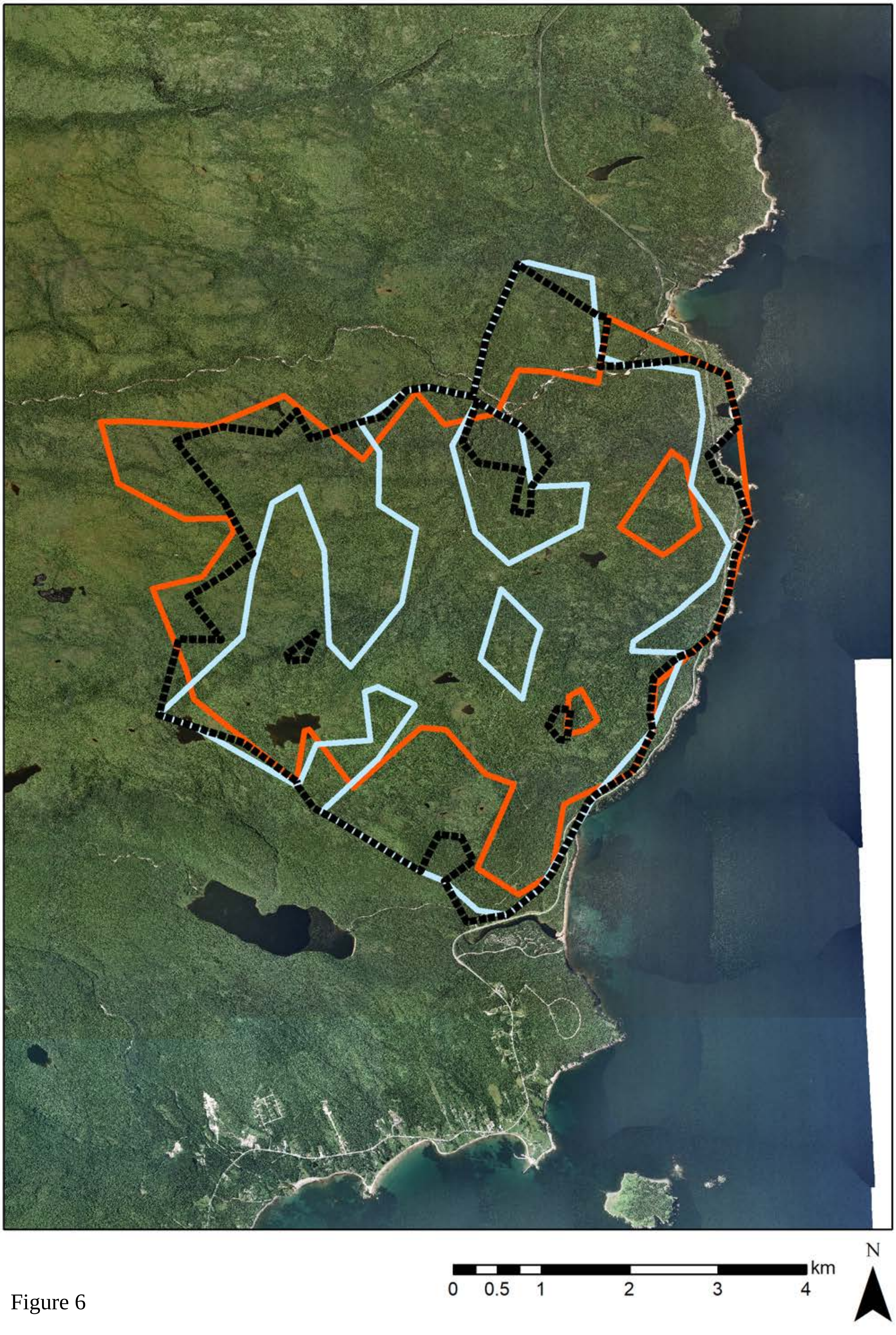

Figure 6

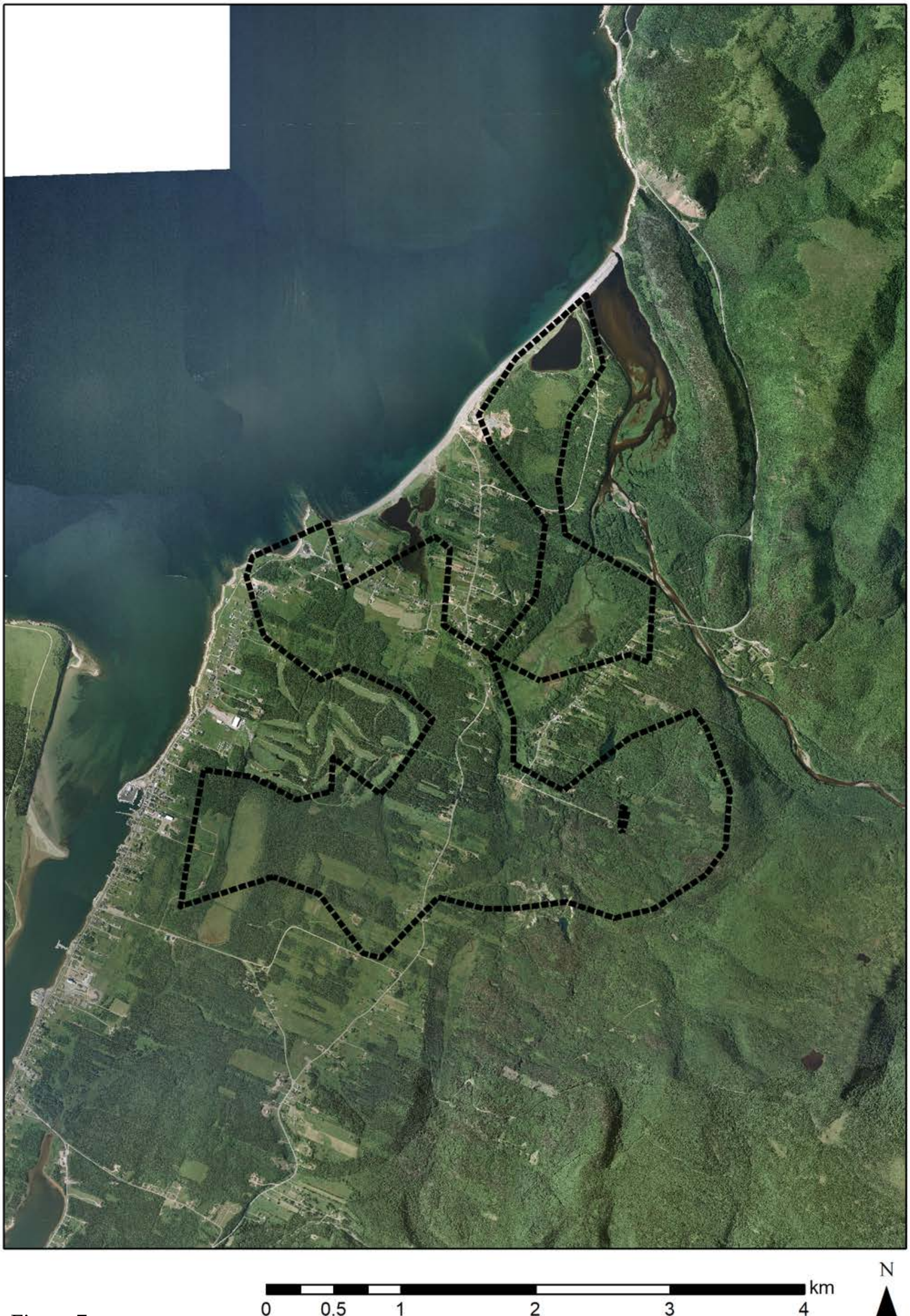

Figure 7

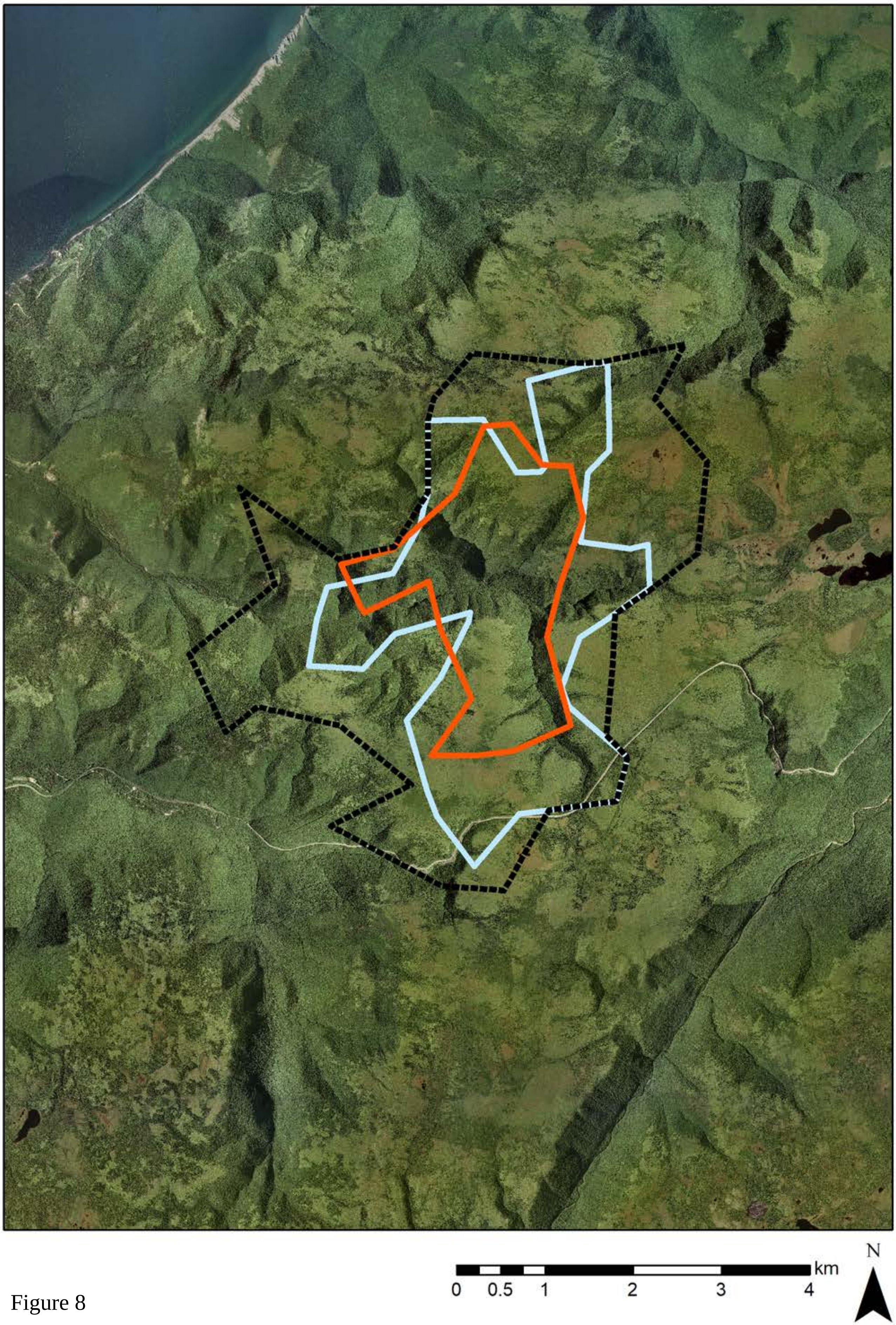

Figure 8

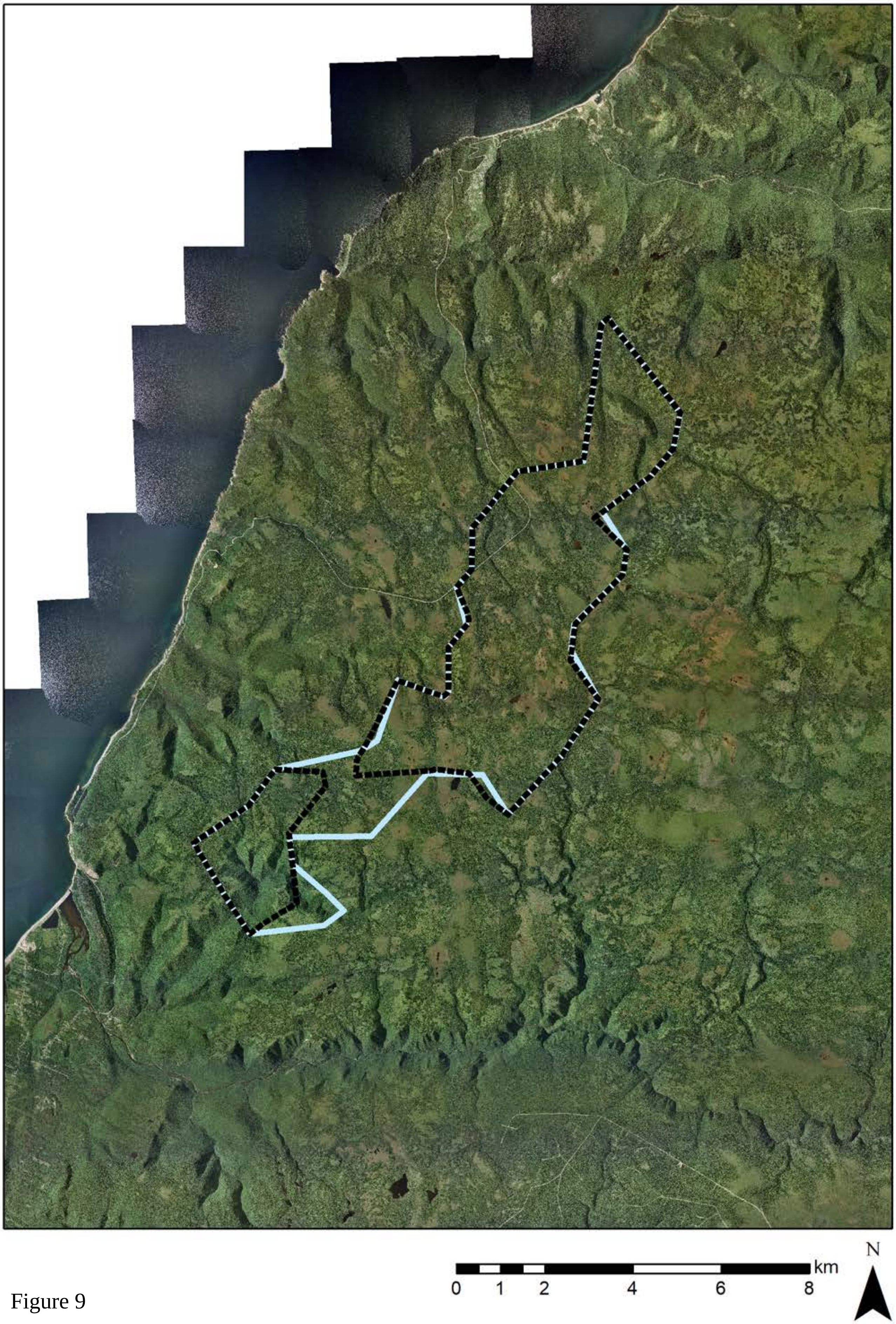

Figure 9

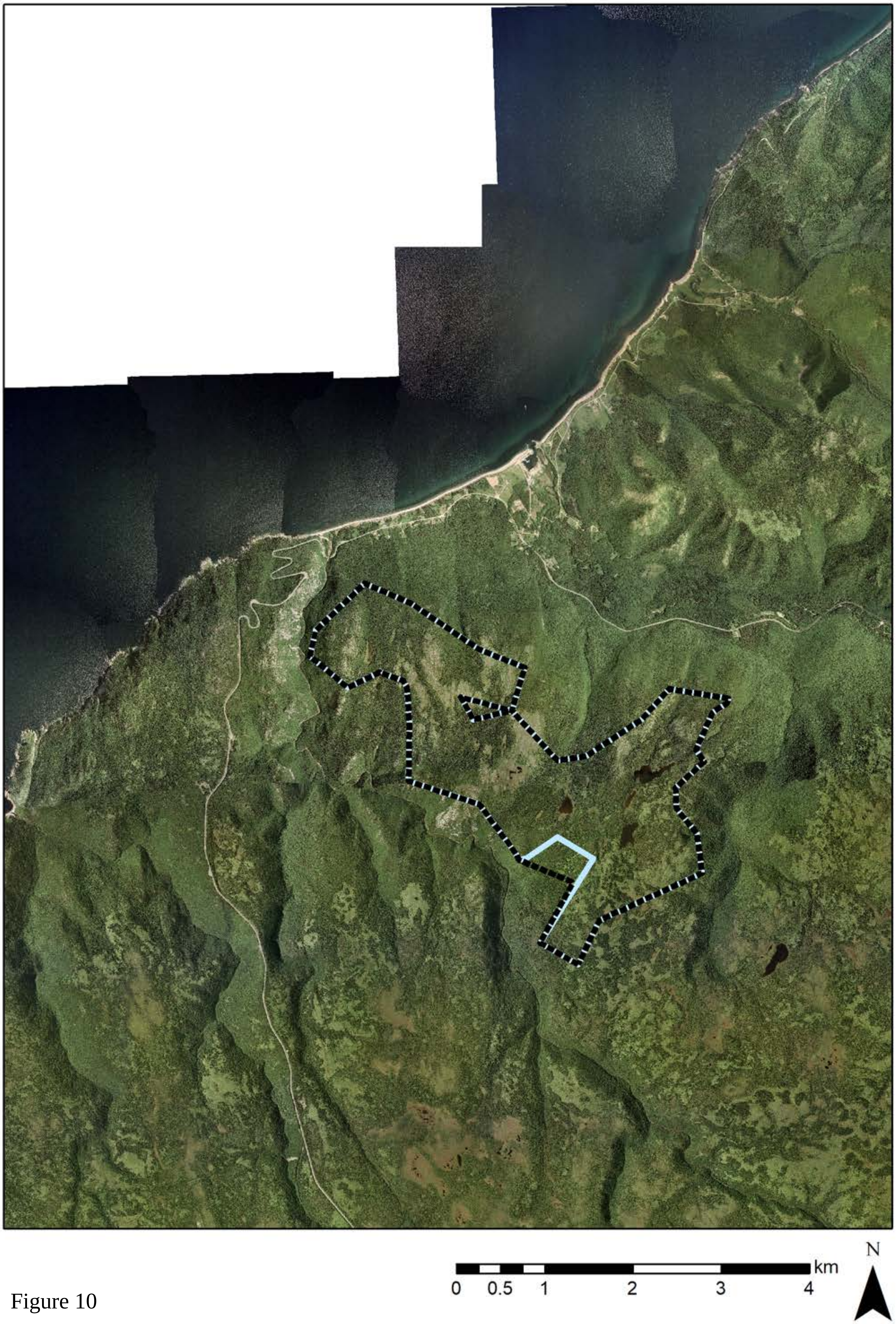

Figure 10

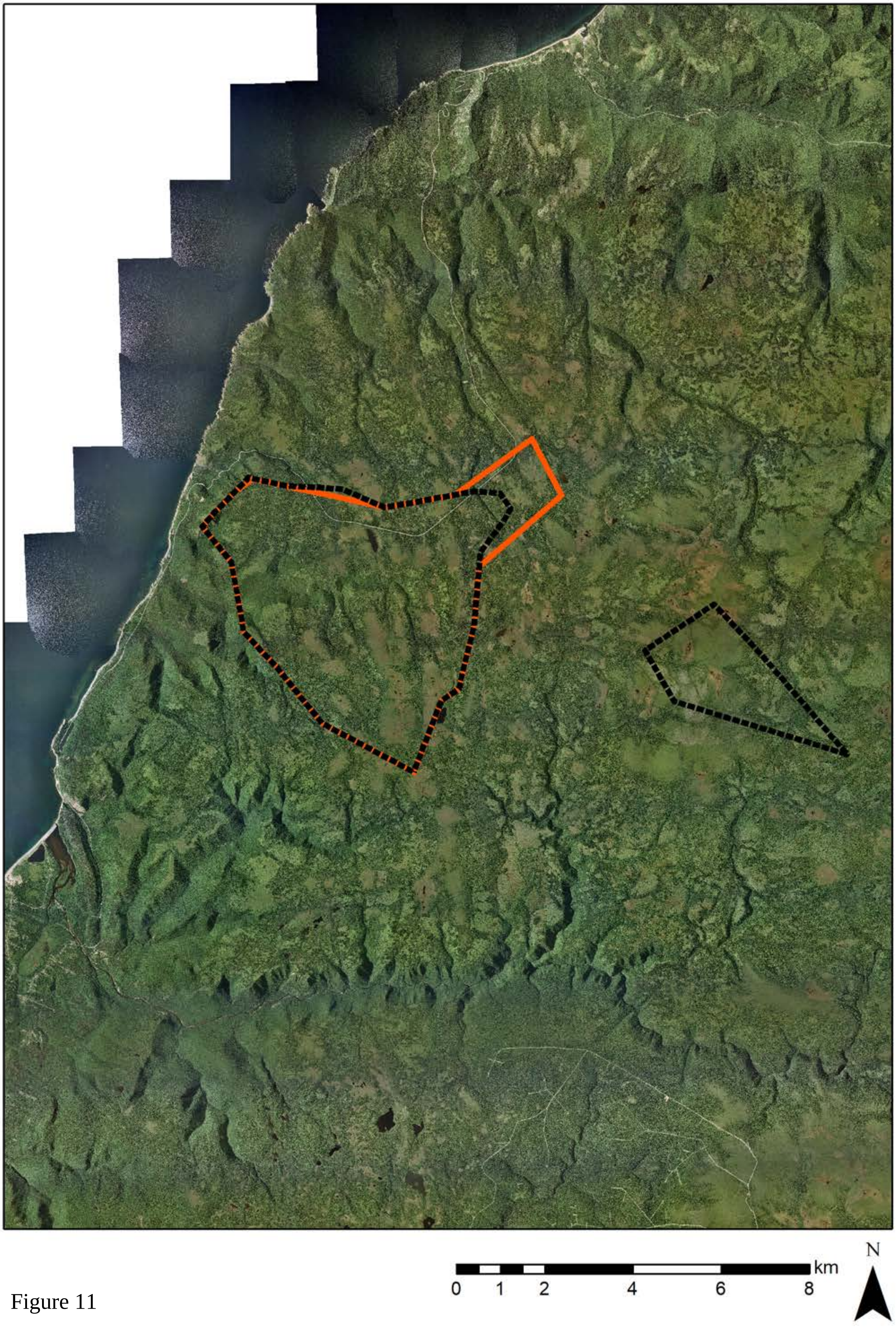

Figure 11

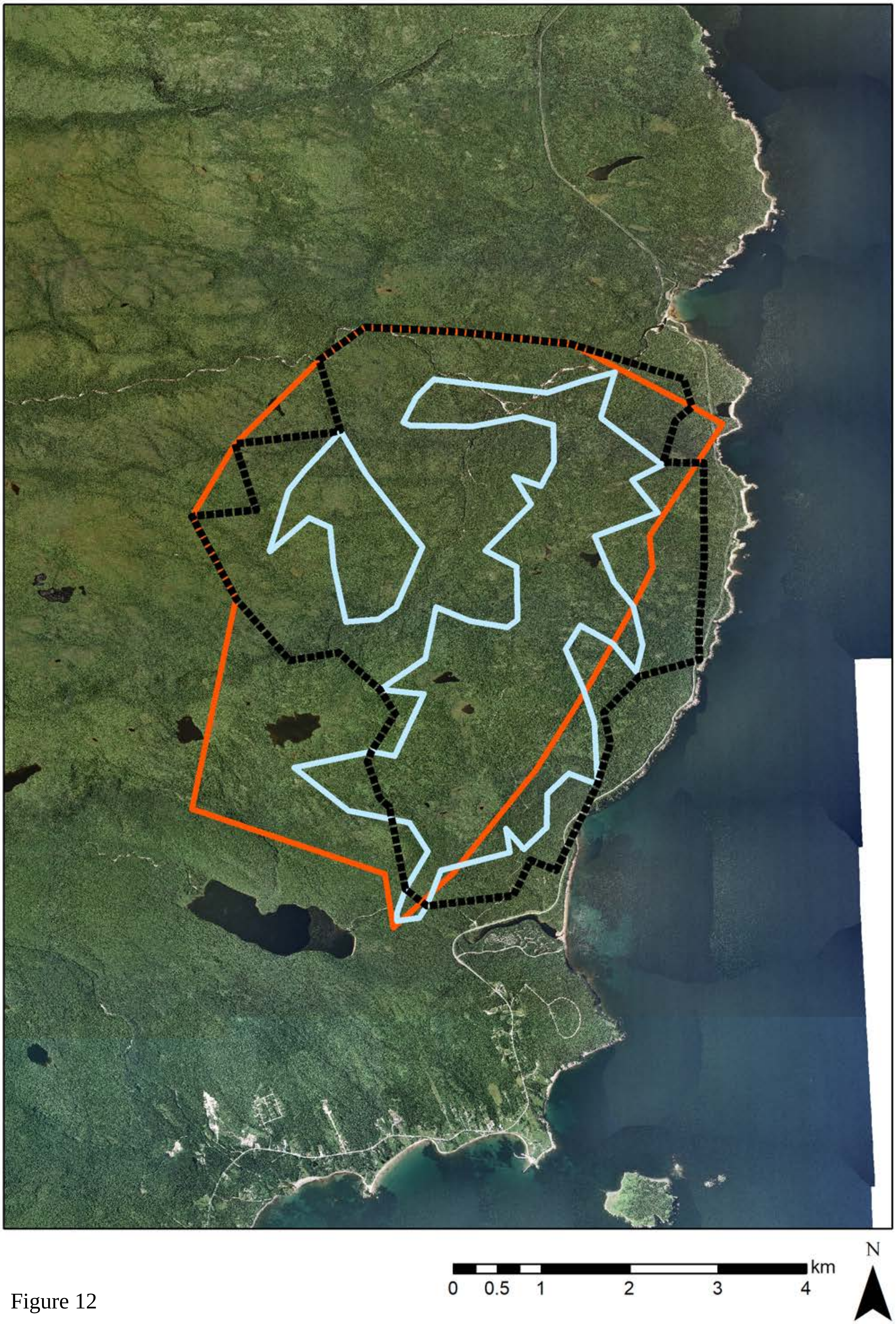

Figure 12

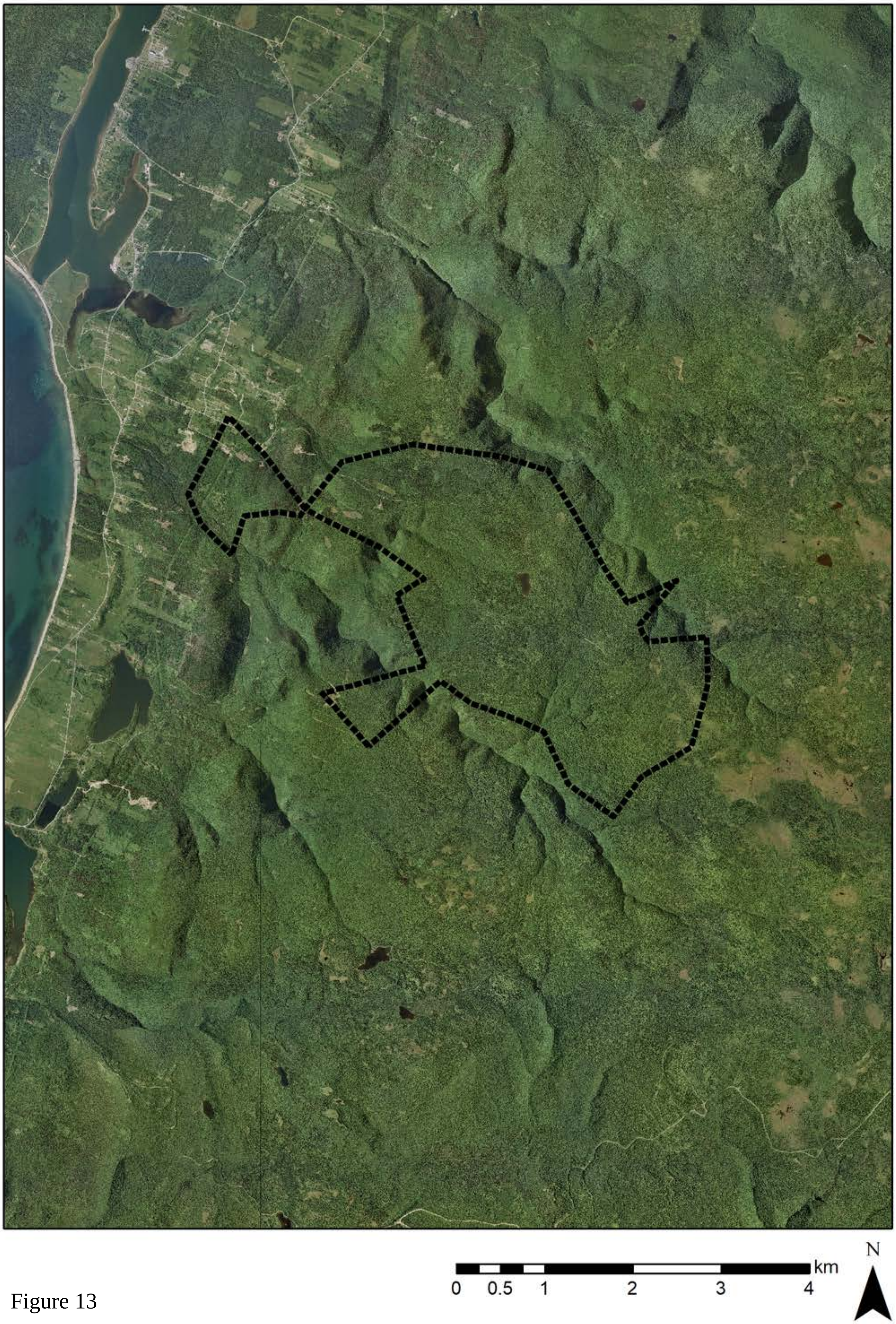

Figure 13

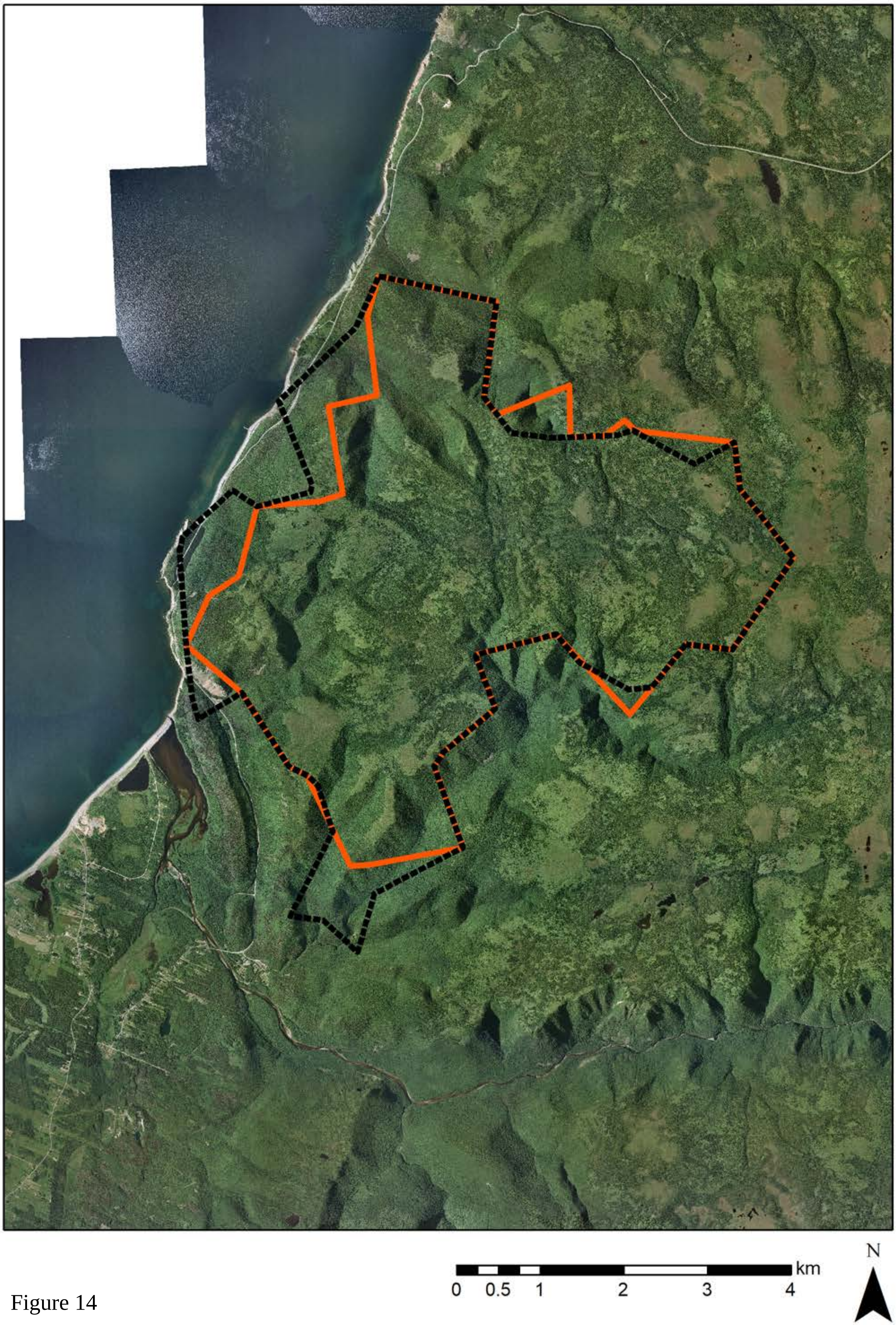

Figure 14

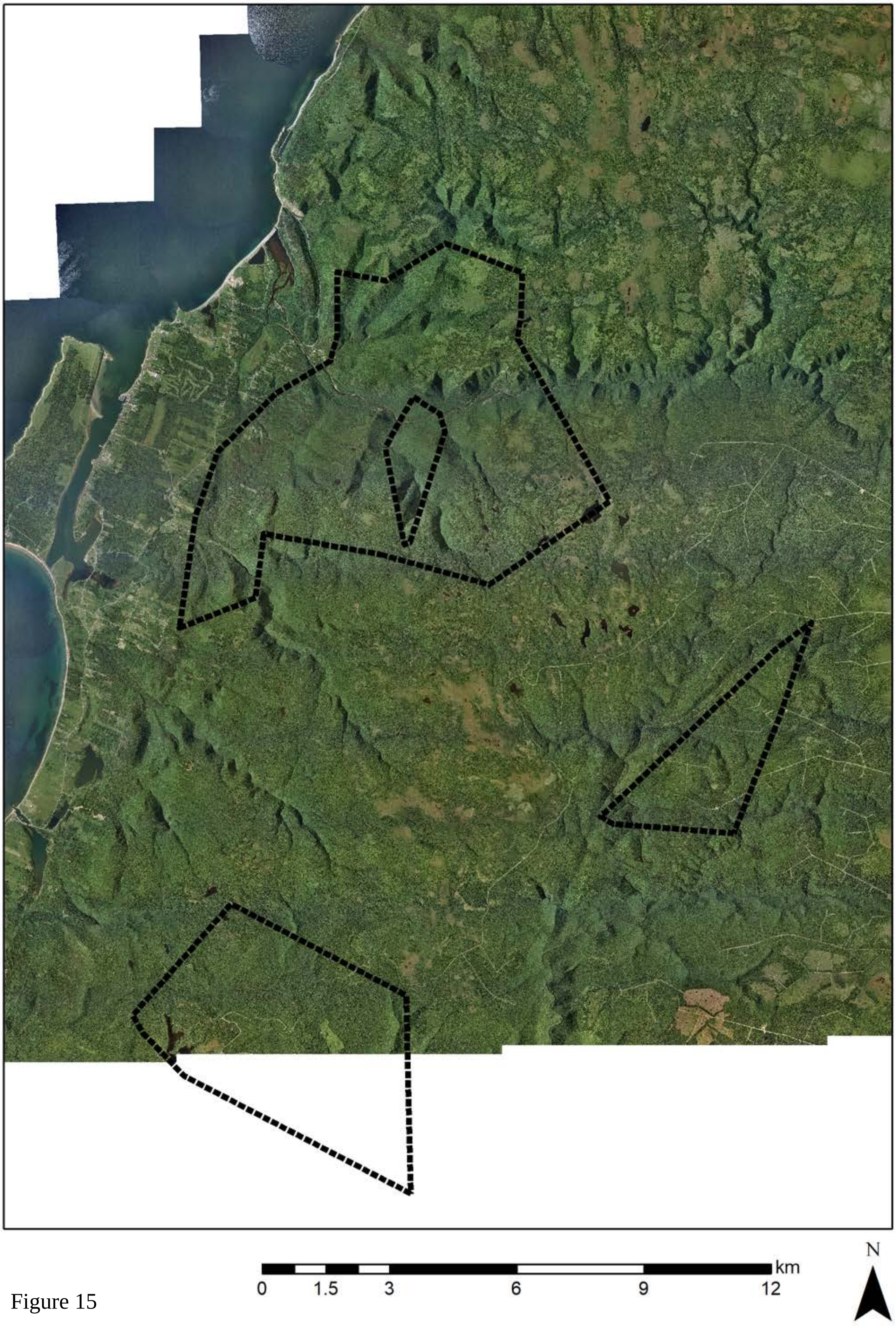

Figure 15

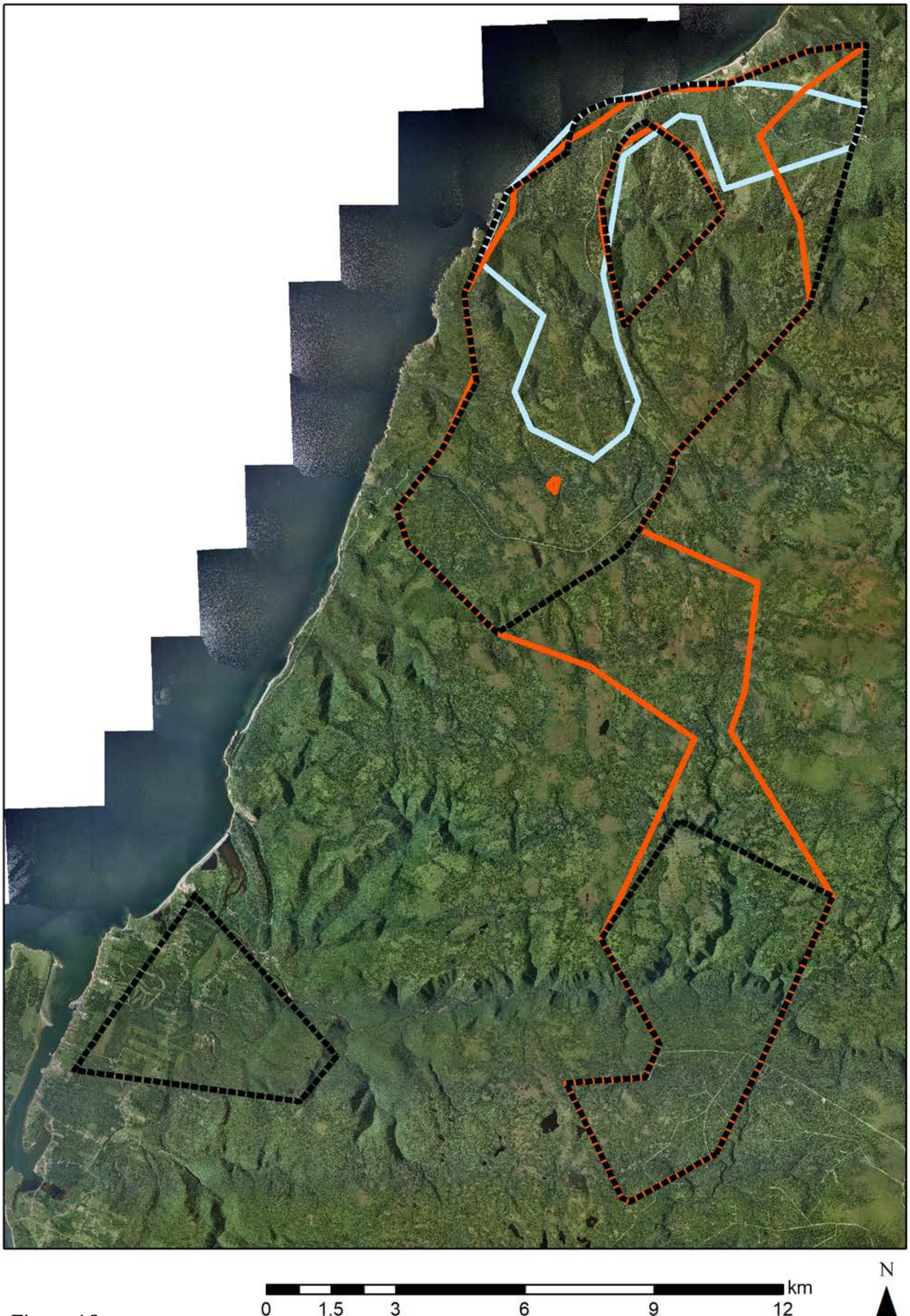

Figure 16

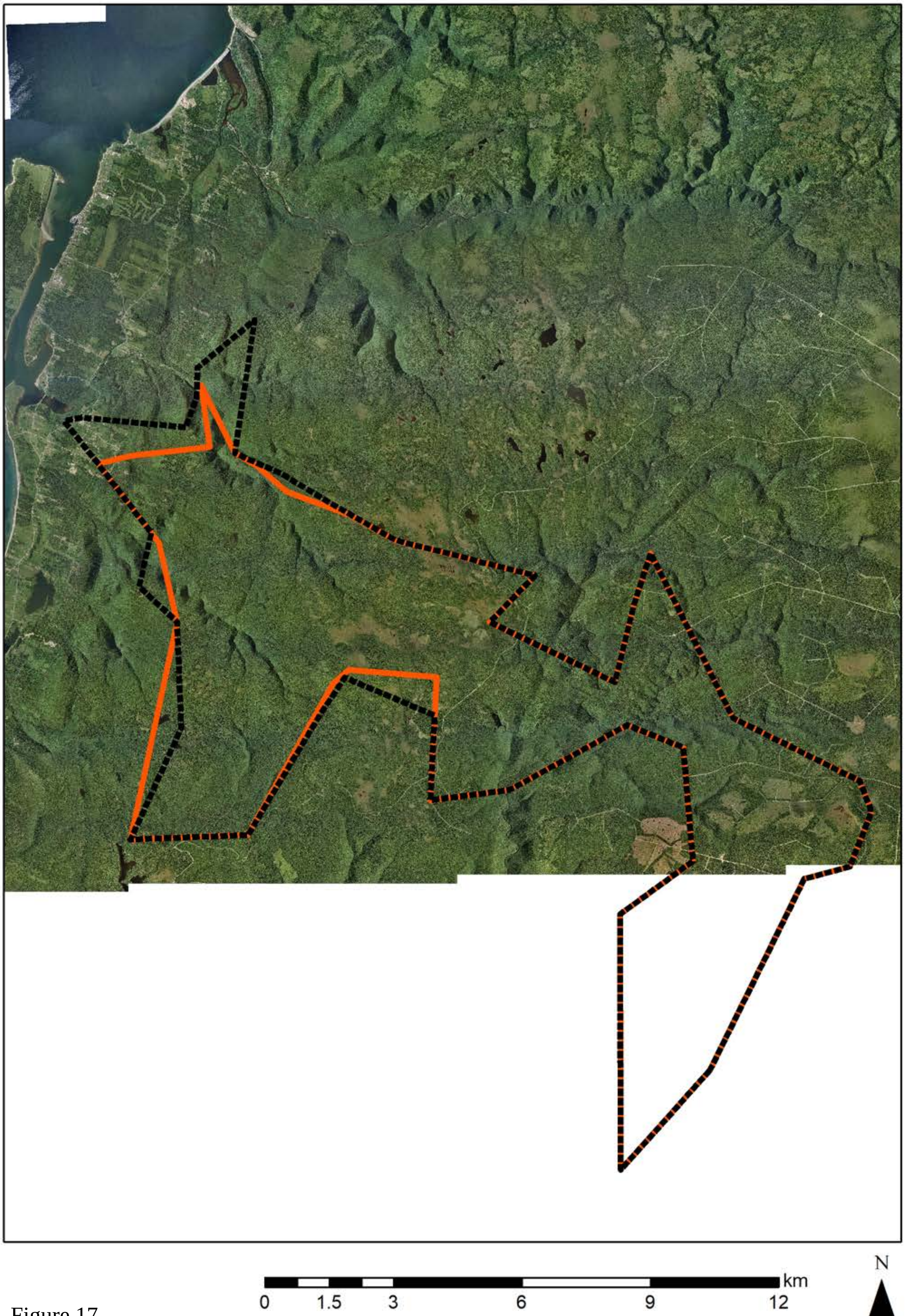

Figure 17

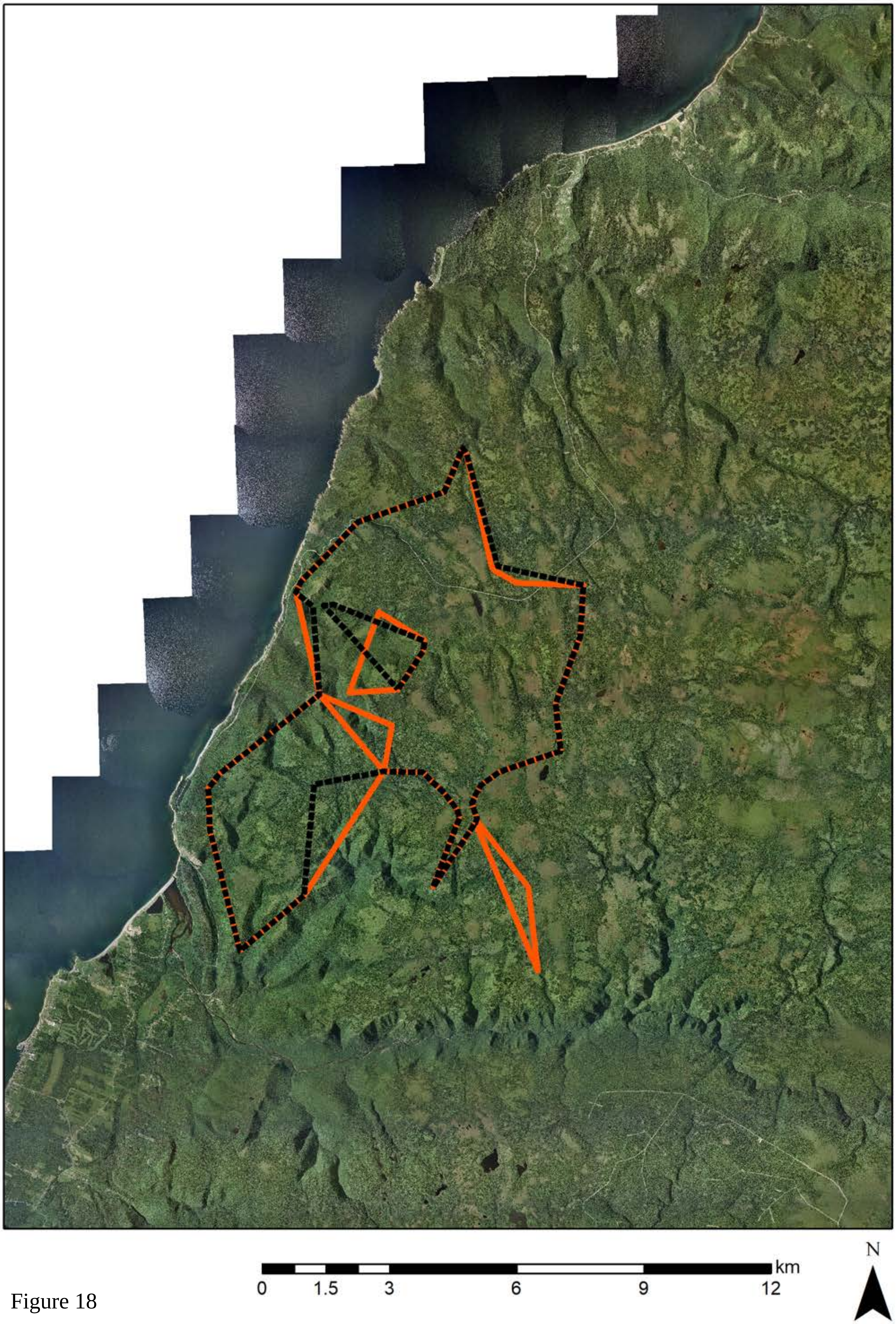

Figure 18

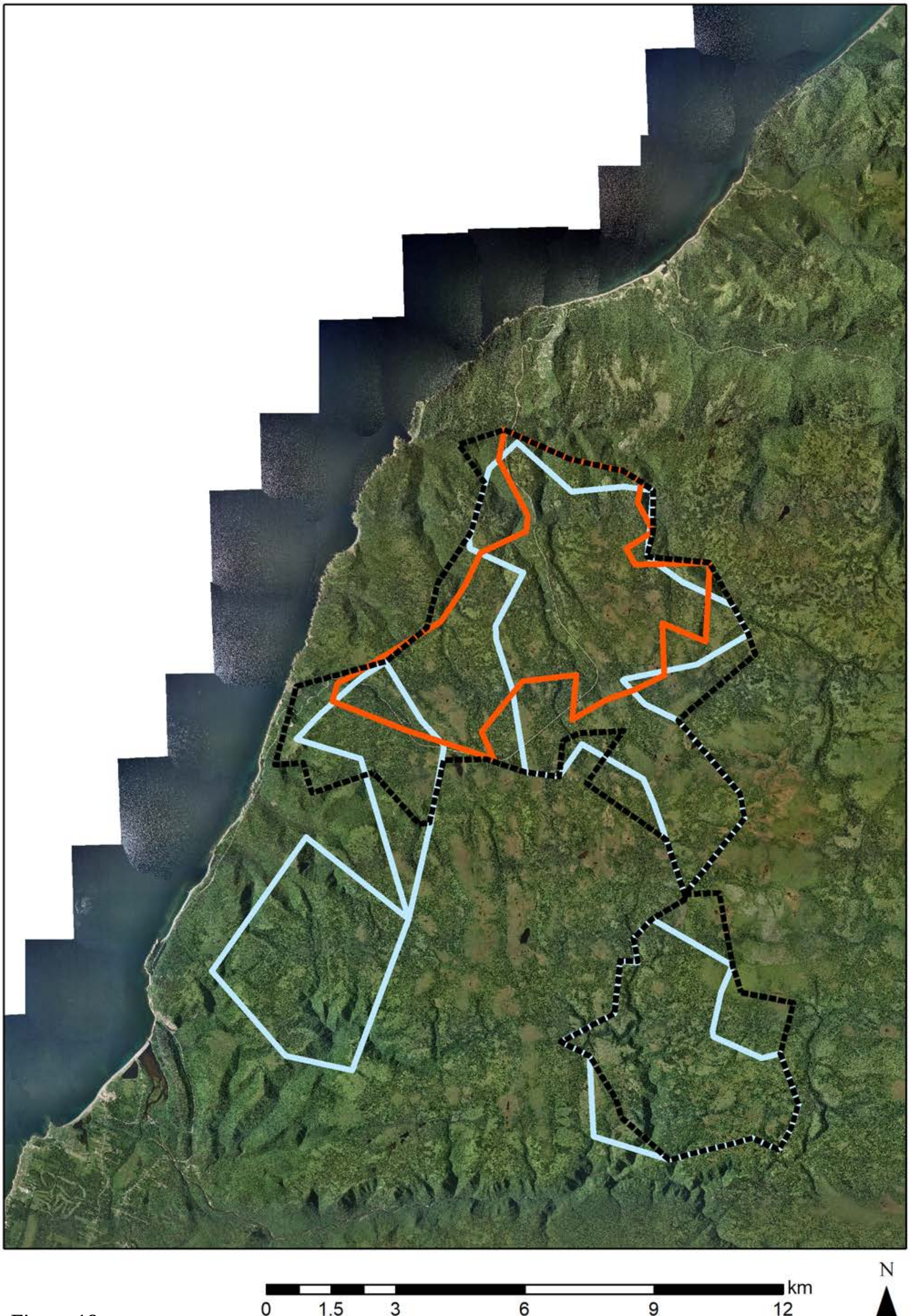

Figure 19

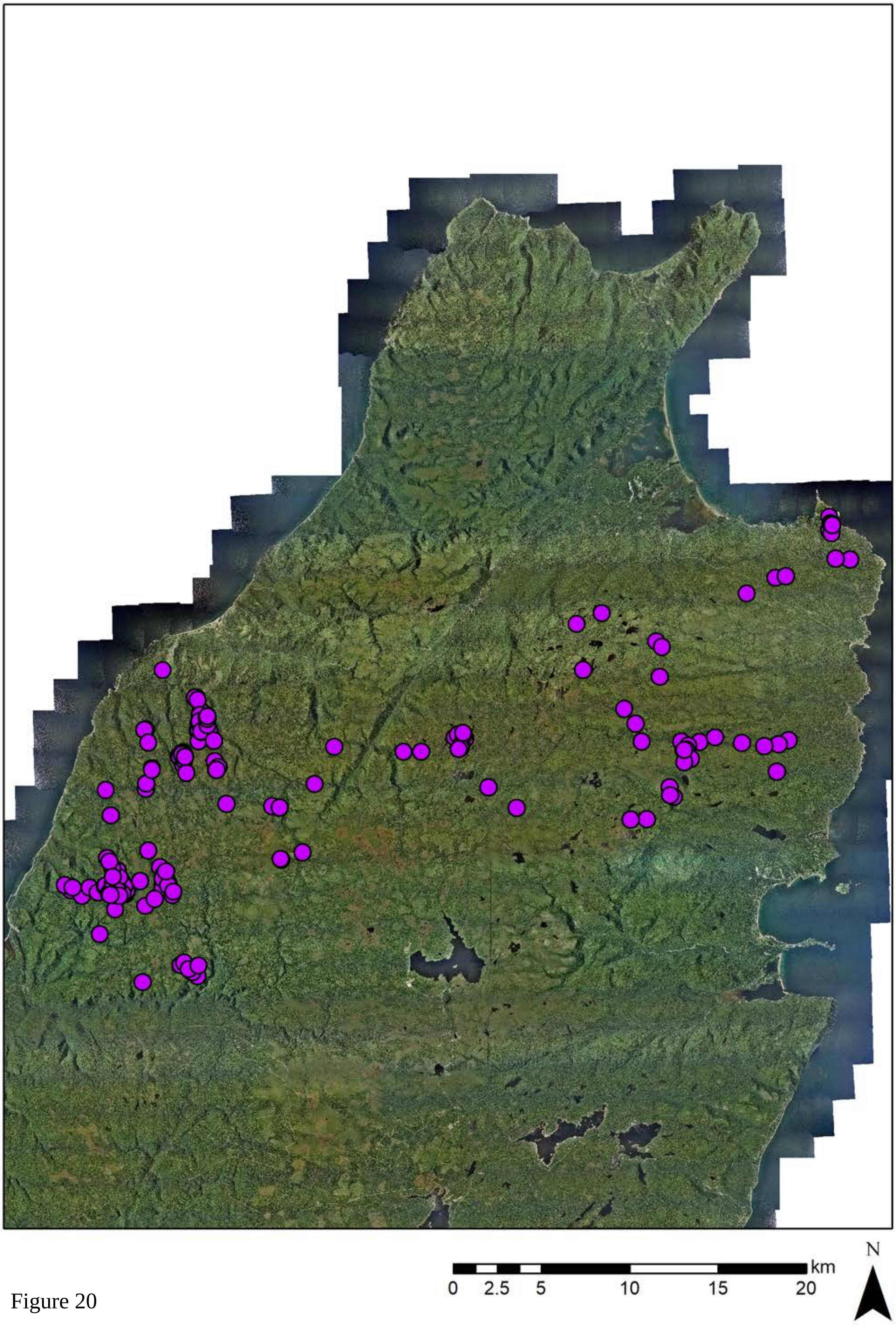

Figure 20

Appendix 3. Results from two-, three-, and four-state movement models

We attempted to categorize coyote (*Canis latrans*) movement behavior using hidden Markov models (HMM) to estimate movement states from movement characteristics (step length and turning angle). We assessed the biologically plausibility and predictive power of models with 2, 3, and 4 movement states. We also compared the fit of these three models to the coyote movement dataset.

Biologically plausible

While the priors we applied to HMM models were based on expected movement parameters of biologically plausible states (2-state: encamped and moving; 3-state: encamped, foraging, and traveling; 4-state: encamped, foraging, searching, traveling), the actual final model and observed parameters did vary. Both the 2-state and 3-state HMM models produced biologically plausible movement states (Table 1 and 2). However, the 4-state HMM model did not produce four biologically distinct movement states. Movement state 2 and 3 (our expected foraging and searching movement states) were not distinct. The step lengths of the two states were very similar (333m (SD = 324m) and 498m (SD = 448) respectively; Table 3). Furthermore, the angle concentration of movement state 3 was unrealistically high (concentration = 1.00; Table 3 and Figure 3). Together, these characteristics led to movement state 3 being very rare in our dataset (27 of 9854; proportion < 0.01; Table 3). As such, we only report on 3 movement states for coyotes in CBHNP: encamped, foraging, and traveling.

Predictive power

All three HMM models had strong predictive ability: the average probability of the predicted state was 0.95 (SD = 0.11) for the 2-state HMM model and was 0.86 (SD = 0.15) and 0.87 (SD = 0.14) for the 3- and 4-state HMM models.

Model selection

As a third assessment criteria, we compared all three HMM models using AIC. Here we found that the 4-state model had the most support (AIC = 177747.9), followed by the 3-state model (AIC = 181168.9), and then the 2-state model (AIC = 182182.7). However, given that the 4-state model did not produce four biologically distinct movement behaviors and the 3rd movement state within the 4-state model was extremely rare, we concluded that the 3-state model was the best model for coyote movement behavior given our data.

Table 1. The model parameters, initial distributions, predicted distributions, and observed movement characteristics of the two movement states in the 2-state Hidden Markov Model used to estimate coyote (*Canis latrans*) movement behavior from 2-hr fix rate data collected from October 2011 to October 2015 in Cape Breton Highlands National Park, Nova Scotia, Canada.

	Encamped	Moving
Initial distribution	0.55	0.45
Predicted distribution	0.35	0.65
Step parameters	7.7m (SD = 5.7m)	805m (SD = 952m)
Angle parameters	3.02 (concentration = 0.27)	-0.01 (concentration = 0.22)
Predicted step characteristics	7.4m (SD = 5.8m; Range = 0.3m – 37m)	873m (SD = 969m; Range = 0.3m – 8619m)

Table 2. The model parameters, initial distributions, predicted distributions, and observed movement characteristics of the three movement states in the 3-state Hidden Markov Model used to estimate coyote (*Canis latrans*) movement behavior from 2-hr fix rate data collected from October 2011 to October 2015 in Cape Breton Highlands National Park, Nova Scotia, Canada.

	Encamped	Foraging	Traveling
Initial distribution	0.52	0.25	0.23
Predicted distribution	0.33	0.32	0.35
Step parameters	6.9m (SD = 4.7m)	215m (SD = 248m)	1331m (SD = 981m)
Angle parameters	3.00 (conc. = 0.27)	-0.07 (conc. = 0.07)	< 0.01 (conc. = 0.32)
Predicted step characteristics	6.6m (SD = 4.6m; Range 0.3m – 29m)	198m (SD = 202m; Range = 0.3m – 1297m)	1426m (SD = 1011m; Range = 59m – 8619m)

Table 3. The model parameters, initial distributions, predicted distributions, and observed movement characteristics of the four movement states in the 4-state Hidden Markov Model used to estimate coyote (*Canis latrans*) movement behavior from 2-hr fix rate data collected from October 2011 to October 2015 in Cape Breton Highlands National Park, Nova Scotia, Canada.

	Encamped	Foraging	Searching	Traveling
Initial distribution	0.51	0.24	0.01	0.24
Predicted distribution	0.34	0.42	< 0.01	0.24
Step parameters	7.0m (SD = 4.9m)	343m (SD = 398m)	479m (SD = 402m)	1705m (SD = 1030m)
Angle parameters	3.00 (conc. = 0.26)	-0.09 (conc. = 0.02)	< -0.01 (conc. = 1.00)	< -0.01 (conc. = 0.27)
Predicted step characteristics	6.8m (SD = 4.8m; Range = 0.3m – 29m)	333m (SD = 324m; Range = 0.3m – 1854m)	498m (SD = 448m; Range = 10m – 1823m)	1803m (SD = 1019m; Range = 214m – 8619m)

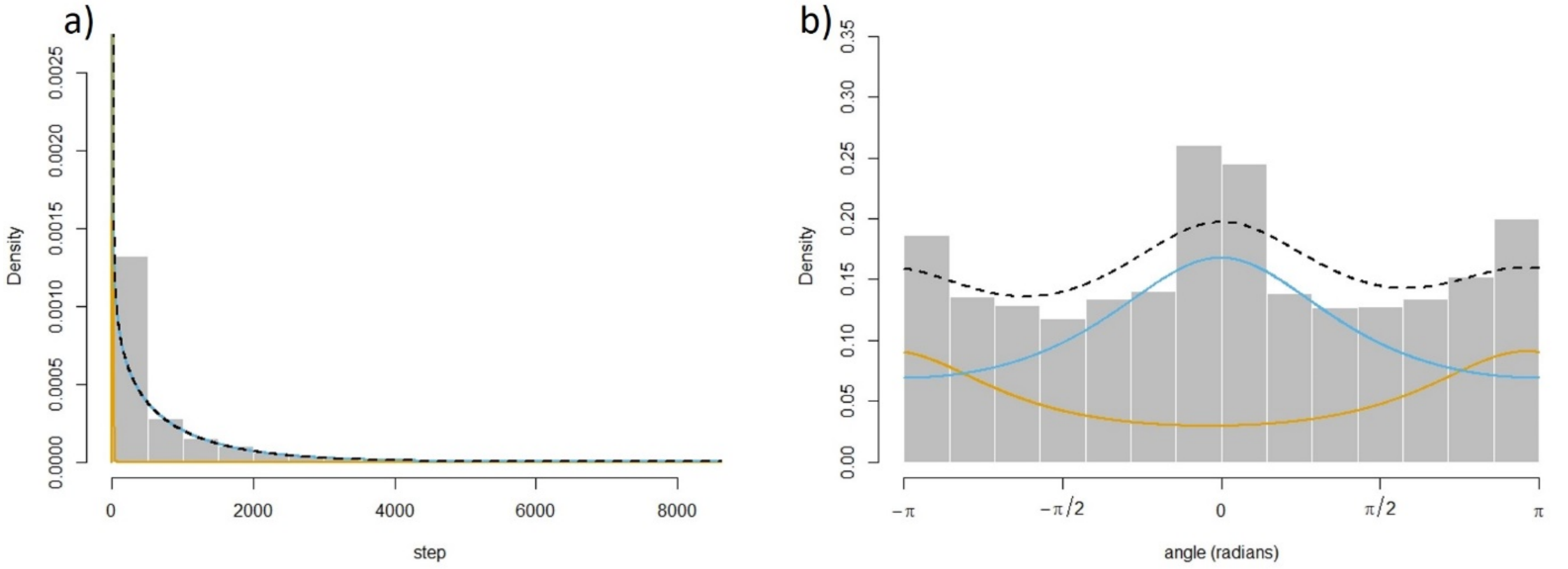

Figure 1. Distribution of step-lengths (a) and turning angles (b) for the encamped (orange) and moving (blue) movement behaviors of coyotes (*Canis latrans*) as estimated using a 2-state hidden Markov model of 2-hr fix rate data collected from October 2011 to October 2015 in Cape Breton Highlands National Park, Nova Scotia, Canada. Complete distribution is shown by the dashed black line.

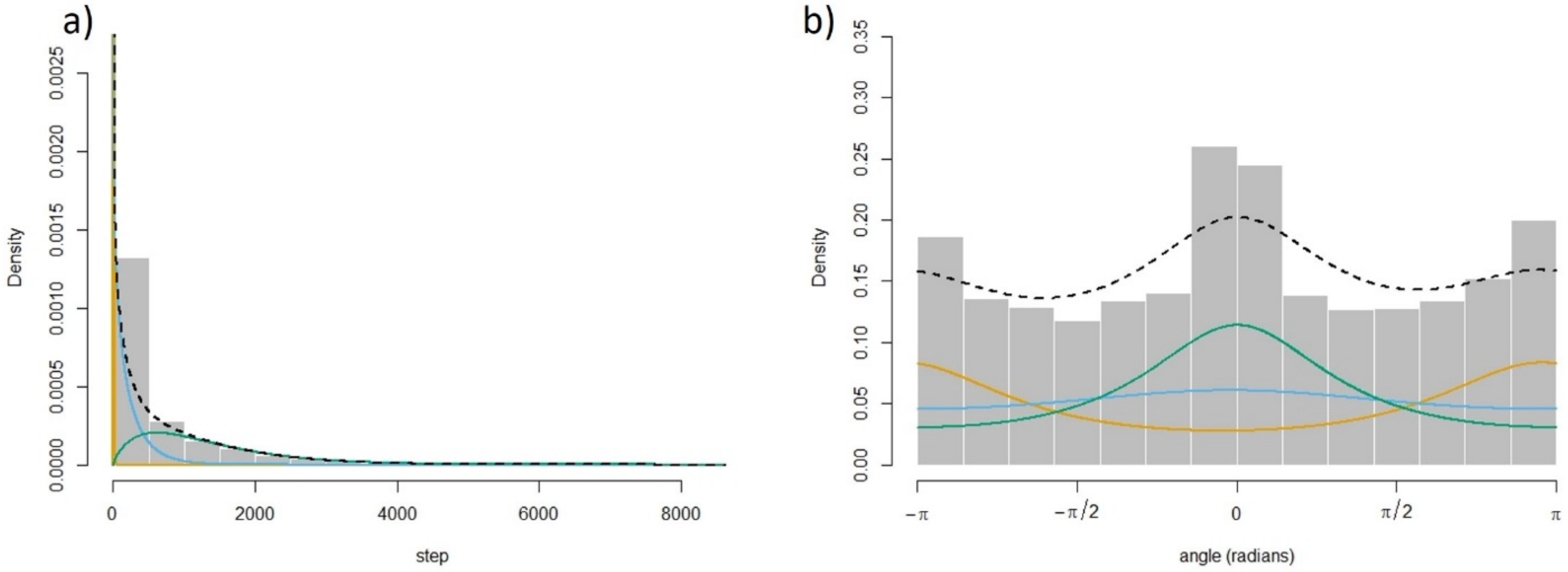

Figure 2. Distribution of step-lengths (a) and turning angles (b) for the encamped (orange), foraging (blue), and traveling (green) movement behaviors of coyotes (*Canis latrans*) as estimated using a 3-state hidden Markov model of 2-hr fix rate data collected from October 2011 to October 2015 in Cape Breton Highlands National Park, Nova Scotia, Canada. Complete distribution is shown by the dashed black line.

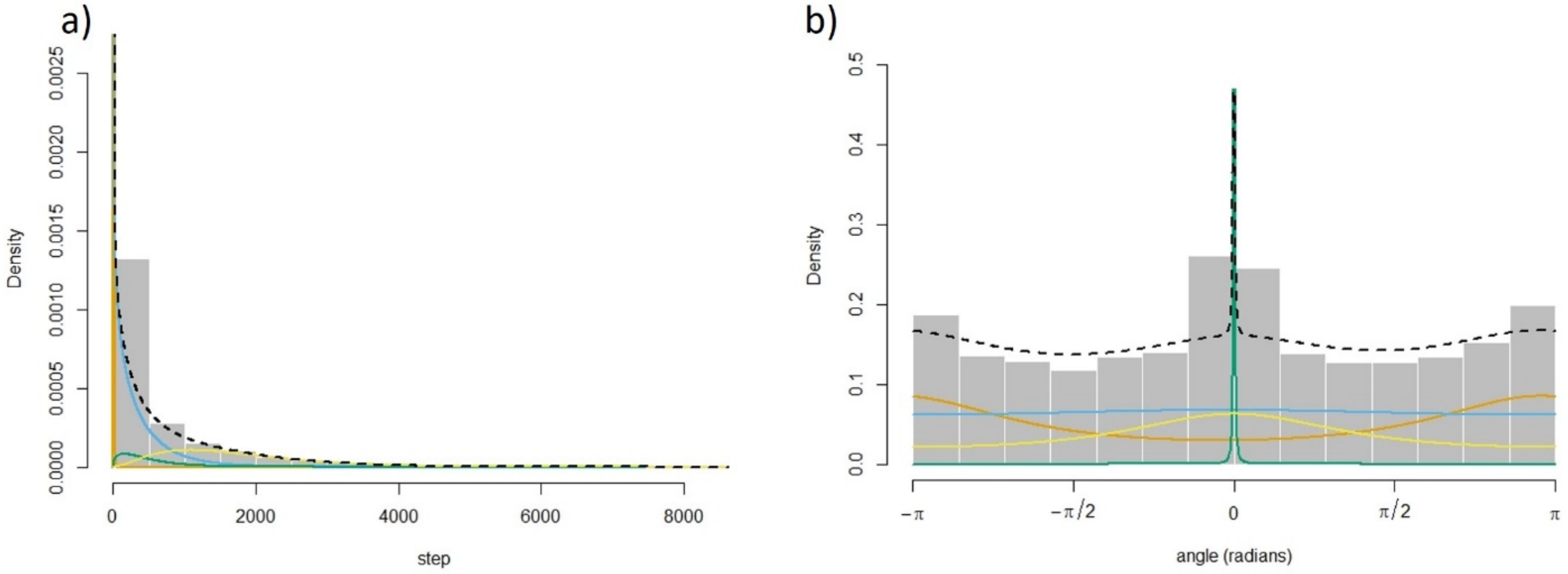

Figure 3. Distribution of step-lengths (a) and turning angles (b) for the encamped (orange), foraging (blue), searching (green), and traveling (yellow) movement behaviors of coyotes (*Canis latrans*) as estimated using a 4-state hidden Markov model of 2-hr fix rate data collected from October 2011 to October 2015 in Cape Breton Highlands National Park, Nova Scotia, Canada. Complete distribution is shown by the dashed black line.
